# Cleavage‐Resistant CYLD Protects Against Autoimmune Hepatitis

**DOI:** 10.1002/advs.202513015

**Published:** 2026-01-28

**Authors:** Han Liu, Chen Su, Jianling Liu, Mingyan Xing, Xiaoxia Wu, Lingxia Wang, Xiaoming Zhao, Hanwen Zhang, YangYang Xie, YangYang Wang, Hong Li, Yu Li, Ming Li, Haibing Zhang

**Affiliations:** ^1^ CAS Key Laboratory of Nutrition Metabolism and Food Safety Shanghai Institute of Nutrition and Health University of Chinese Academy of Sciences Chinese Academy of Sciences Shanghai P. R. China; ^2^ CAS Key Laboratory of Computational Biology Shanghai Institute of Nutrition and Health Chinese Academy of Sciences Shanghai P. R. China; ^3^ State Key Laboratory of Protein and Plant Gene Research School of Life Sciences Peking University Beijing P. R. China

**Keywords:** autoimmune hepatitis, CYLD, immune‐mediated hepatitis, MEK1/2

## Abstract

Autoimmune hepatitis (AIH) is an immune‐mediated liver disease that can progress to fibrosis, cirrhosis, and hepatocellular carcinoma. However, the pathogenic mechanisms underlying AIH remain poorly understood, limiting the development of effective therapies. Here, using a concanavalin A‐induced murine model of experimental autoimmune hepatitis (EAH), proteolytic cleavage of the deubiquitinase cylindromatosis (CYLD) at Asp215 is identified as a critical molecular event that promotes disease progression. Mice harboring a macrophage‐specific, cleavage‐resistant *Cyld^D215A/D215A^* mutation are markedly protected from hepatic injury, indicating that CYLD stability is a key regulator of liver inflammation. Mechanistically, TNFα induces CYLD cleavage in macrophages, which enhances alarmin‐triggered chemokine production through activation of MEK1/2 signaling. Further analyses reveal that CYLD and the E3 ubiquitin ligase TRIM25 cooperatively regulate MEK1/2 ubiquitination at lysine residues K192/K196. MEK1/2 ubiquitination promotes its activation by strengthening its interaction with RAF1 and drives subsequent chemokine production. Importantly, pharmacological inhibition of MEK1/2 significantly attenuates EAH severity. Together, these findings uncover a previously unrecognized CYLD‐MEK1/2 axis in macrophages that orchestrates hepatic inflammation and identify MEK signaling as a potential therapeutic target for AIH.

## Graphical abstract

Proteolytic cleavage of the deubiquitinase CYLD emerges as a critical driver of autoimmune hepatitis. TNFα‐induced CYLD loss in macrophages amplifies S100A9‐triggered MAPK activation, leading to excessive chemokine production and hepatic inflammation. Pharmacological inhibition of MEK signaling effectively attenuates experimental disease, highlighting a tractable therapeutic axis for immune‐mediated liver injury.

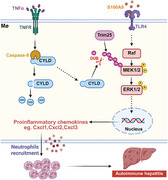

## 1 Introduction

Autoimmune hepatitis (AIH) is a severe immune‐mediated liver disease characterized by progressive necroinflammatory damage, immune cell activation, and infiltration [1]. Despite a rising incidence worldwide, the precise pathogenesis of AIH remains incompletely elucidated [2]. Current understanding points to a complex interplay between genetic susceptibility and environmental triggers (e.g., viral infections, drugs, gut dysbiosis), culminating in dysregulated immunity and chronic hepatic injury [1, 3, 4]. Without intervention, AIH often progresses to cirrhosis and liver failure. While immunosuppressive therapy is the cornerstone of treatment, suboptimal responses and frequent relapses in a substantial patient subset underscore the critical need for deeper mechanistic insights and novel targeted therapeutic strategies [1, 5].

Recent studies have highlighted the central role of post‐translational modifications, particularly ubiquitination, in regulating immune signaling and inflammatory responses. Ubiquitination dynamically modulates the stability, localization, and activity of intracellular proteins and is implicated in diverse liver diseases, including viral hepatitis, steatohepatitis, and hepatocellular carcinoma [6, 7, 8, 9]. Among the enzymes regulating this process, the deubiquitinase CYLD is of particular interest. CYLD specifically removes K63‐linked polyubiquitin chains from key signaling adaptors and kinases, including NEMO, TAK1, RIPK1, and TRAFs, which are integral to NF‐κB and MAPK signaling and the regulation of cell death pathways. By deubiquitinating these molecules, CYLD attenuates downstream pro‐inflammatory and pro‐survival signaling [10, 11, 12, 13, 14, 15]. CYLD has been linked to a range of pathological conditions, including cancer [16, 17, 18, 19, 20, 21, 22], inflammatory disorders [23, 24, 25, 26], and metabolic diseases [27], positioning it as an attractive therapeutic target. While previous studies on CYLD function in liver disease focused primarily on its role in hepatocellular carcinoma [16, 17, 22], acute/chronic injury [28], and non‐alcoholic steatohepatitis (NASH) [27], the contribution of CYLD to AIH remains unexplored.

Alarmins, also known as damage‐associated molecular patterns (DAMPs), are endogenous molecules released upon cellular stress or injury that potently activate innate and adaptive immunity [29, 30]. Currently, the major categories of alarmins include defensins, high‐mobility group (HMG) proteins, interleukins (ILs), heat shock proteins (HSPs), S100 proteins, uric acid [29, 30]. Elevated serum levels of HMGB1 and IL‐33 in AIH patients suggest their potential involvement in disease progression [31]. Although alarmins contribute significantly to autoimmune diseases like systemic lupus erythematosus, rheumatoid arthritis, and inflammatory bowel disease, the role of alarmin signaling in AIH pathogenesis remains poorly defined [32, 33, 34].

To investigate the molecular drivers of AIH, we utilized the concanavalin A (Con A)‐induced experimental autoimmune hepatitis (EAH) model, which recapitulates key features of human AIH [35, 36]. Our study reveals that Caspase‐8‐mediated proteolytic cleavage of CYLD at Asp215 is a previously unrecognized mechanism contributing to EAH progression. Notably, mice expressing a cleavage‐resistant *Cyld^D215A/D215A^* mutant specifically in macrophages exhibited profound protection from liver injury, implicating CYLD stability in macrophages as a critical checkpoint in hepatic inflammation. Mechanistically, we demonstrate that TNFα‐induced CYLD cleavage in macrophages enhances their responsiveness to the alarmin S100A9 through MEK1/2 signaling activation, leading to increased chemokine production and subsequent neutrophil recruitment. Furthermore, we discovered that CYLD, in cooperation with the E3 ubiquitin ligase TRIM25, regulates K63‐linked ubiquitination of MEK1/2 at lysine residues K192/K196. The ubiquitination of MEK1/2 promotes its activation by strengthening its interaction with RAF1, and drives subsequent chemokine production. Importantly, pharmacological inhibition of MEK1/2 significantly ameliorated liver inflammation in the EAH model, suggesting a promising therapeutic strategy for AIH.

Collectively, our findings uncover a novel CYLD‐MEK1/2 regulatory axis in macrophages that drives immune‐mediated liver pathology. By elucidating a previously unappreciated role for CYLD cleavage in EAH, our study provides mechanistic insights into the disease and identifies MEK1/2 as a promising target for therapeutic intervention in autoimmune hepatitis.

## 2 Results

### 2.1 Cleavage‐Resistant CYLD Protects Mice From ConA‐Induced EAH

**FIGURE 1:**
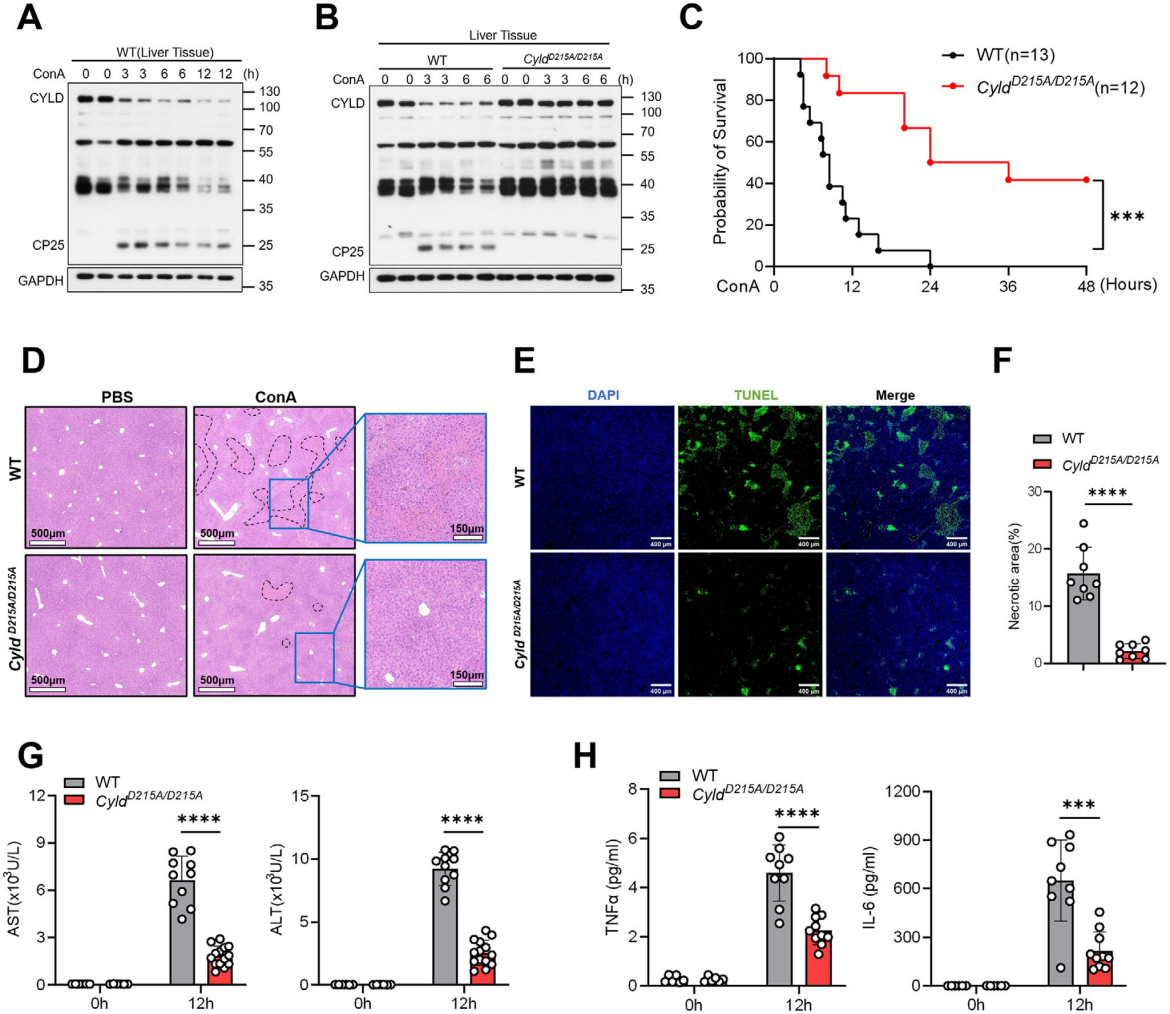
Cleavage‐resistant CYLD D215A mutation protects mice from ConA‐induced EAH. (A) Western blot analysis of CYLD in liver tissues from WT mice intravenously injected with ConA (12 mg/kg, i.v.) for the indicated times. (B) Western blot analysis of CYLD in liver tissues from WT and *Cyld^D215A/D215A^* mice treated with ConA (12 mg/kg, i.v.) for the indicated times. (C) Survival of WT (n=13) and *Cyld^D215A/D215A^* (n=12) mice after injection with ConA (25 mg/kg, i.v.). Statistical significance was analyzed using the log‐rank (Mantel‐Cox) test. (D–H) Mice of the indicated genotypes were injected with ConA (12 mg/kg, i.v.). Liver tissues and serum were collected 12 h post‐injection (n ≥ 6 mice per group). Representative images of (D) H&E staining and (E) TUNEL staining of liver sections are shown. (F) Quantification of TUNEL‐positive areas. Serum levels of (G) aminotransferases (AST and ALT) and (H) cytokines (IL‐6 and TNFα) were analyzed. Statistical analysis for panels (F–H) was performed using a two‐tailed unpaired Student's *t*‐test. Data are presented as mean ± SD. *p < 0.05, **p < 0.01, ***p < 0.001, ****p < 0.0001.

To investigate the role of CYLD in AIH, we induced EAH in wild‐type (WT) mice through ConA intravenous injection and examined CYLD protein levels in the liver. Immunoblot analysis revealed a marked reduction of full‐length CYLD protein during EAH progression, accompanied by the appearance of a 25‐kDa N‐terminal cleavage fragment (termed CP25) (Figure 1A). Notably, the decrease in full‐length CYLD protein was evident as early as 3 h after ConA challenge and became more pronounced at later time points.

To determine whether the reduction of CYLD was regulated at the transcriptional or post‐translational level, we quantified Cyld mRNA expression in liver tissues following ConA treatment. Reverse transcription quantitative PCR (RT‐qPCR) analysis showed that Cyld mRNA levels were not suppressed, but instead transiently upregulated after ConA stimulation before gradually returning toward baseline (Figure S1). These findings indicate that the rapid loss of CYLD protein during EAH is not due to transcriptional downregulation, but is predominantly regulated at the post‐translational level.

Previous studies have established that under stimulation by TNFα, LPS, or FasL, full‐length CYLD undergoes Caspase‐8‐mediated cleavage at Asp215(D215), leading to decreased intracellular levels of intact CYLD and generation of an N‐terminal 25‐kDa fragment [37, 38, 39]. To determine whether CYLD reduction in EAH was due to cleavage at this site, we generated *Cyld^D215A/D215A^* mice, in which the Caspase‐8 cleavage site (D215) was mutated to alanine (A215), rendering CYLD resistant to proteolytic processing. Upon ConA‐induced EAH, *Cyld^D215A/D215A^* mice exhibited no significant reduction in hepatic CYLD levels, and the CP25 fragment was completely absent (Figure 1B), confirming that CYLD depletion in this disease model is primarily dependent on Caspase‐8‐dependent cleavage at D215.

The strong correlation between cleavage‐induced CYLD degradation and EAH progression prompted us to investigate whether cleavage‐resistant CYLD could confer protection against disease development. Indeed, *Cyld^D215A/D215A^* mice displayed remarkable resistance to EAH compared with WT controls, as evidenced by significantly improved survival rates (Figure 1C). Histopathological analysis revealed a significant decrease in the degree of liver necrosis in mutant mice (Figure 1D–F), along with markedly lower serum levels of aspartate aminotransferase (AST) and alanine aminotransferase (ALT) (Figure 1G). Furthermore, *Cyld^D215A/D215A^* mice exhibited attenuated inflammatory responses, with significantly decreased serum TNFα and IL‐6 levels following ConA challenge (Figure 1H). Collectively, these findings establish a critical role for caspase‐8–mediated CYLD cleavage in EAH pathogenesis and highlight the protective effect of cleavage‐resistant CYLD in autoimmune liver injury.

### 2.2 Macrophage‐Specific CYLD Cleavage Resistance Confers Protection in EAH

**FIGURE 2:**
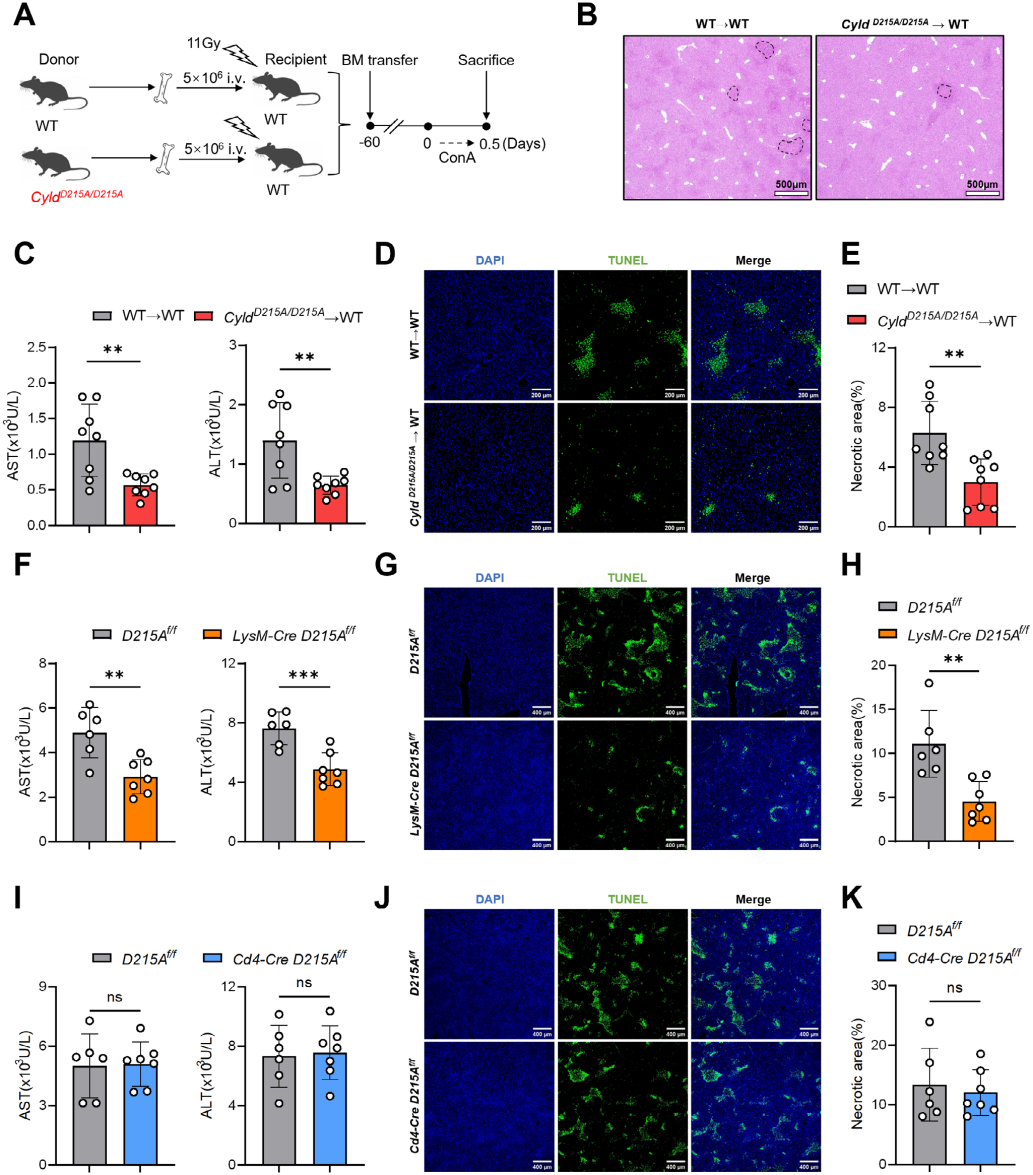
CYLD D215A in macrophages is sufficient to confer protection against EAH. (A–E) Bone marrow transplantation (BMT) experiments: BM cells from WT or *Cyld^D215A/D215A^* mice were transplanted into lethally irradiated (11 Gy) recipient mice. After 8 weeks of reconstitution, EAH was induced by intravenous ConA injection (12 mg/kg; 12 h) (n = 8 mice per group). (A) Experimental design schematic. (B) Representative H&E‐stained liver sections (scale bar: 500 µm). (C) Serum AST and ALT levels (U/L). (D) TUNEL staining of liver sections (scale bar: 200 µm). (E) Quantification of TUNEL‐positive areas. (F–K) Mice of the indicated genotypes were injected with ConA (12 mg/kg; 12 h). (n ≥ 6 mice per group). (F,I) Serum AST and ALT levels (U/L). (G,J) TUNEL staining of liver sections (scale bar: 400 µm). (H,K) Quantification of TUNEL‐positive areas. Statistical analysis for panels (C,E,F,H,I,K) was performed using a two‐tailed unpaired Student's *t*‐test. Data are presented as mean ± SD, *p < 0.05, **p < 0.01, ***p < 0.001; ns, not significant.

To determine which cell population mediates the protective effect of CYLD cleavage resistance on D215 in EAH, we performed bone marrow transplantation (BMT) experiments (Figure 2A). When recipient mice received hematopoietic stem cells (HSCs) from *Cyld^D215A/D215A^* donors, they exhibited significant protection against EAH, as evidenced by reduced serum AST and ALT levels and a diminished degree of liver necrosis (Figure 2B‐E). However, when *Cyld^D215A/D215A^* recipient mice were reconstituted with WT HSCs, their resistance to EAH was abolished, and they even displayed exacerbated liver injury (Figure S2A–E). These findings collectively indicate that the protective effect in EAH is mediated by hematopoietic lineage cells.

To further identify the cell type responsible for CYLD cleavage‐dependent protection in EAH, we generated conditional *Cyld^D215A^* knock‐in mice *Cyld ^loxp‐WT‐stop‐loxp‐D215A^* (hereafter referred to as *D215A^f/f^*) (Figure S3A,B). Given the established roles of T cells and macrophages in AIH pathogenesis [1], we crossed *D215A^f/f^* mice with *Cd4‐Cre* and *LysM‐Cre* strains to generate T cell– and macrophage‐specific CYLD D215A mutants, respectively. Importantly, cell‐type–specific sequencing analysis confirmed that the D215A substitution occurred selectively in the intended target cell populations in each conditional mouse line (Figure S3C).

Following ConA challenge, *LysM‐Cre; D215A^f/f^* mice exhibited significantly lower AST and ALT levels (Figure 2F) and a reduced degree of liver necrosis (Figure 2G,H; Figure S2F). In contrast, *Cd4‐Cre; D215A^f/f^* mice showed no significant difference in liver injury compared to controls (Figure 2I–K; Figure S2G). These results demonstrate that macrophage‐specific CYLD cleavage resistance at D215 is sufficient to confer protection against EAH.

### 2.3 Cleavage‐Resistant CYLD in Macrophages Suppresses Neutrophil‐Recruiting Chemokine Production

**FIGURE 3:**
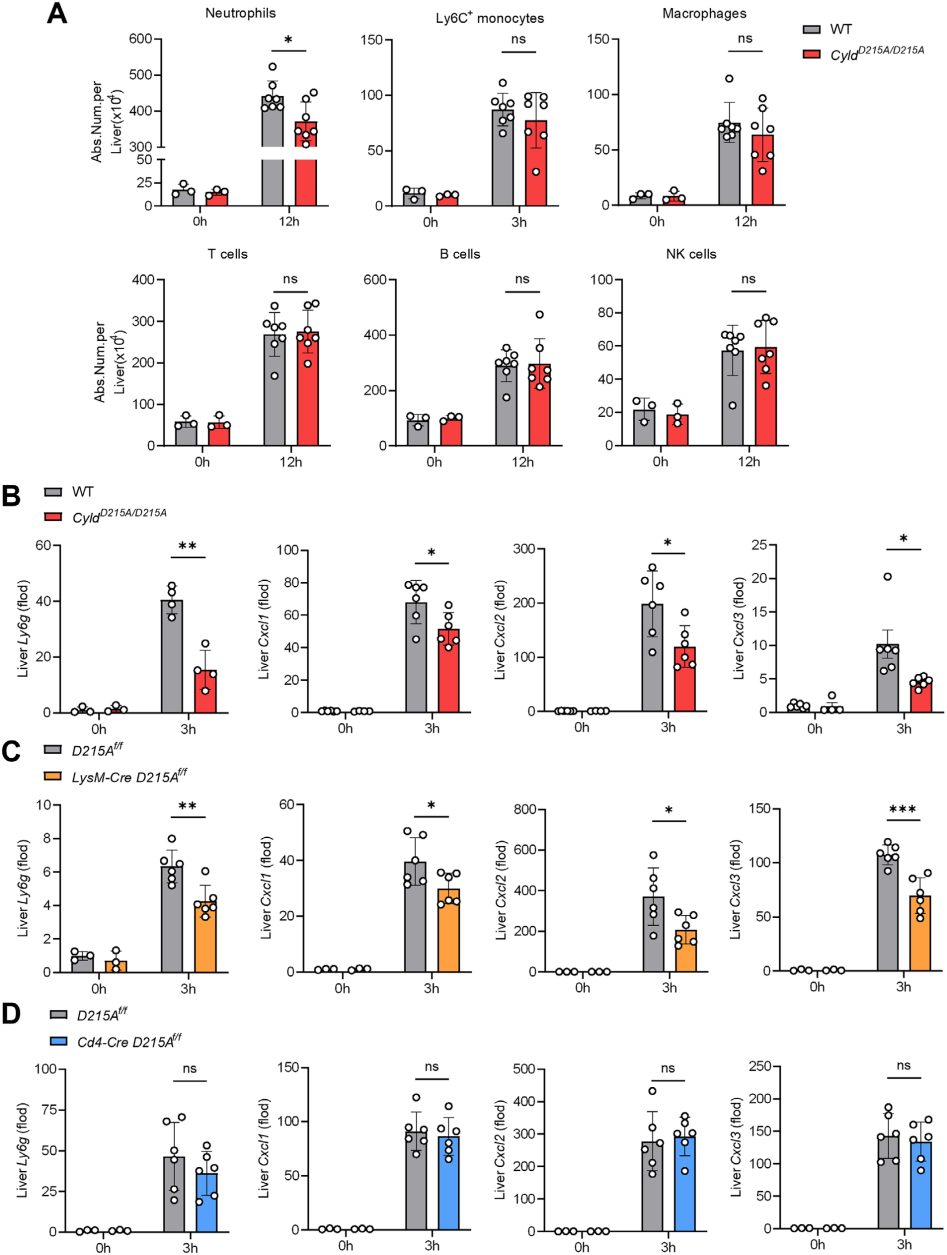
Macrophage CYLD D215A attenuates EAH by suppressing neutrophil‐recruiting chemokine production. (A) Flow cytometry analysis of liver immune cells from mice of the indicated genotypes after ConA treatment (5 mg/kg, i.v.) (n = 3 mice per group for untreated controls and n = 7 mice per group for ConA‐treated groups). (B–D) RT‐qPCR analysis of neutrophils marker gene (*Ly6g*) and neutrophil‐recruiting chemokines genes (*Cxcl1, Cxcl2*, and *Cxcl3*) in liver tissues from mice of the indicated genotypes at 3 h after ConA treatment (12 mg/kg, i.v.) (n = 3 mice per group for untreated controls and n ≥ 4 mice per group for ConA‐treated groups). Statistical analysis was performed using a two‐tailed unpaired Student's *t*‐test. Data are presented as mean ± SD, *p < 0.05, **p < 0.01, ***p < 0.001; ns, not significant.

To elucidate how CYLD cleavage contributes to EAH progression, we further analyzed the immune profiles of *Cyld^D215A/D215A^* mice in ConA‐induced EAH. Flow cytometry revealed that *Cyld^D215A/D215A^* mice exhibited markedly reduced neutrophil accumulation in the liver, without alterations in monocyte, macrophage, NK cell, T cell, or B cell populations (Figure 3A). A parallel reduction in splenic neutrophils was observed (Figure S4A). Consistent with these findings, *Cyld^D215A/D215A^* mice livers demonstrated significantly lower expression of neutrophil‐associated genes (Figure 3B; Figure S4B). Given that neutrophil recruitment is primarily governed by chemokines (e.g., Cxcl1/2/3) [40, 41], we quantified hepatic chemokine levels and found pronounced reductions in neutrophil‐recruiting chemokines in *Cyld^D215A/D215A^* mice (Figure 3B; Figure S4C), while lymphocyte and monocyte‐recruiting chemokines remained unchanged (Figure S4D), which was consistent with the flow cytometry data.

We employed conditional mutant mice to determine the type of leukocytes that was producing lower Cxcl1/2/3 in *Cyld^D215A/D215A^* mice. Macrophages are the major source of Cxcl1/2/3 neutrophil‐recruiting chemokines [42, 43, 44]. We found that *LysM‐Cre; D215A^f/f^* mice exhibited significantly decreased neutrophil accumulation and reduced neutrophil‐recruiting chemokines expression in EAH (Figure 3C), whereas *Cd4‐Cre; D215A^f/f^* mice exhibited no differences compared to controls (Figure 3D). Given that the LysM promoter is also active in neutrophils, we next assessed neutrophil responsiveness to chemotactic stimulation. Neutrophils from *LysM‐Cre; D215A^f/f^* mice displayed comparable responses to chemokines relative to controls (Figure S4E,F), indicating that the reduced neutrophil accumulation observed in vivo is unlikely to result from impaired neutrophil chemokine responsiveness.

These findings indicate that macrophages carrying the cleavage‐resistant CYLD D215A mutation play a key role in neutrophil‐recruiting chemokines expression in ConA‐induced EAH, rather than T cells or neutrophils themselves. These results are consistent with our previous conclusion that the protective effect is mediated by macrophages rather than T cells.

### 2.4 TNFα Induces CYLD Cleavage in Macrophages During EAH

**FIGURE 4:**
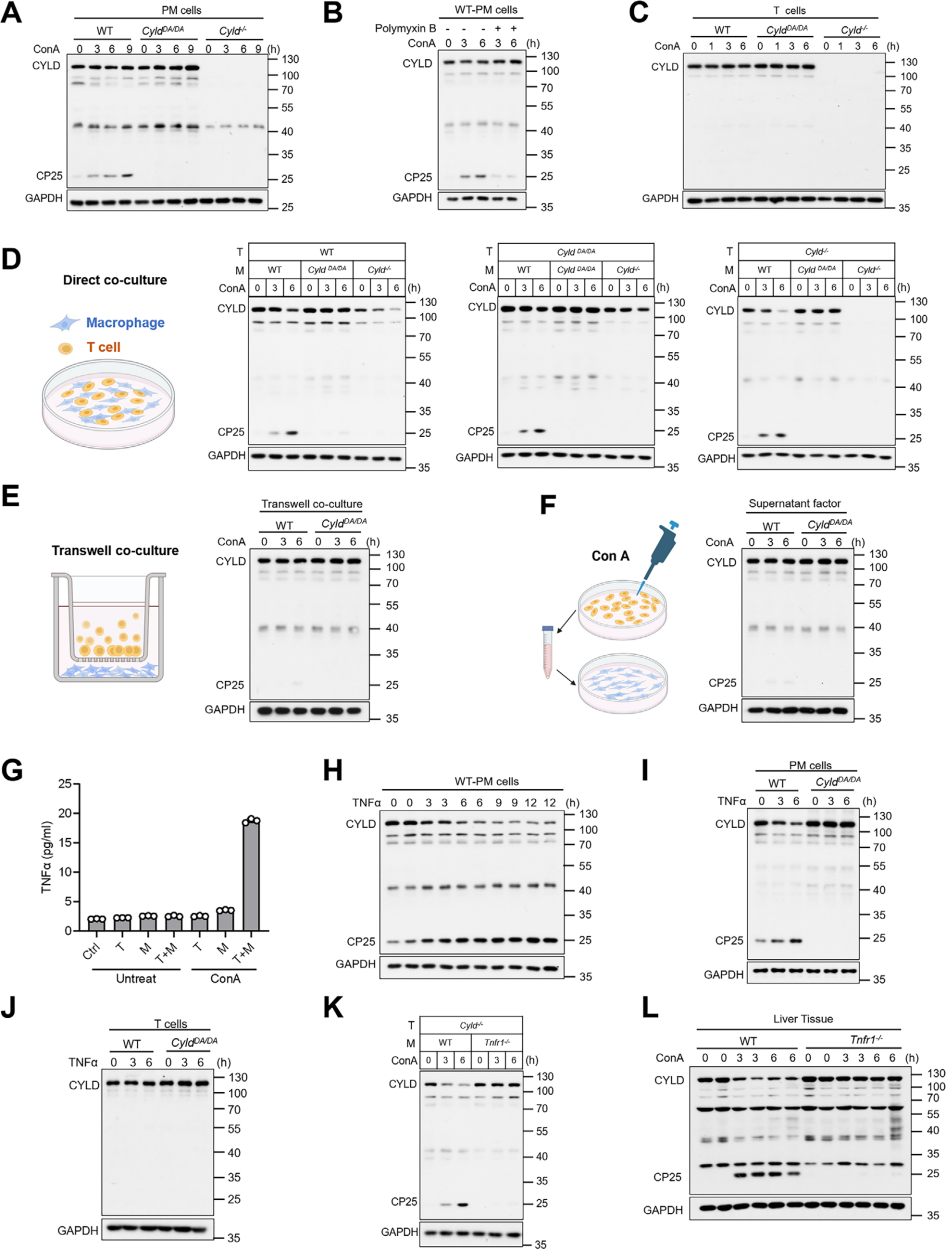
T‐cell contact triggers TNFα‐mediated CYLD cleavage in macrophages during EAH. (A) Western blot analysis of peritoneal macrophages (PMs) from WT, *Cyld^−/−^* and *Cyld^D215A/D215A^* (*Cyld^DA/DA^*) mice stimulated with ConA (10 µg/mL) for the indicated durations. (B) WT PMs were stimulated with ConA (10 µg/mL) ± Polymyxin B (25 µg/mL) for the indicated durations. (C) Splenic CD3^+^ T cells were treated with ConA (10 µg/mL) and Polymyxin B (25 µg/mL) for the indicated durations. (D) Schematic illustration and experimental validation of co‐culture of splenic CD3^+^ T cells and PMs with the indicated genotype combinations, stimulated with ConA (10 µg/mL) and Polymyxin B (25 µg/mL) for the indicated durations; mixed cell lysates were analyzed by immunoblotting. (E) Schematic illustration and experimental validation of WT splenic CD3^+^ T cells co‐cultured with PMs of the indicated genotypes using a Transwell system (PMs in the lower chamber and T cells in the upper chamber), followed by stimulation with ConA (10 µg/mL) and Polymyxin B (25 µg/mL) for the indicated durations; PMs were harvested for immunoblot analysis. (F) Schematic illustration and experimental validation of WT splenic CD3^+^ T cells were stimulated with ConA (10 µg/mL) and Polymyxin B (25 µg/mL) for 6 h. Culture supernatants were collected and used to treat PMs of the indicated genotypes for the specified durations, followed by immunoblot analysis of PM lysates. (G) ELISA analysis of TNFα secretion in culture supernatants from WT splenic CD3^+^ T cells alone (T), WT PMs alone (M), and WT T cells co‐cultured with WT PMs (T+M), either untreated or treated with ConA (10 µg/mL) and Polymyxin B (25 µg/mL) for 9 h, with RPMI‐1640 medium serving as the control (Ctrl). (H–J) (H, I) PMs or (J) splenic CD3^+^ T cells of the indicated genotypes were stimulated with mouse TNFα (20 ng/mL) for the indicated times. (K) Co‐culture of *Cyld^−/−^* splenic CD3^+^ T cells and PMs with the indicated genotypes, followed by stimulation with ConA (10 µg/mL) and Polymyxin B (25 µg/mL) for the indicated times; mixed cell lysates were analyzed by immunoblotting. (L) Western blot analysis of CYLD in liver tissues from WT and *Tnfr1^−/−^* mice after ConA injection (12 mg/kg, i.v.).

The observation that prevention of CYLD cleavage in macrophages protected mice from ConA‐induced EAH prompted us to investigate the factors regulating CYLD cleavage in macrophages during EAH. Given that ConA has been reported to directly activate macrophages [45, 46, 47], we examined the effect of ConA stimulation on macrophage activation status. Direct ConA treatment led to increased expression of multiple inflammatory mediators in macrophages (Figure S5A). We next examined whether ConA could directly induce CYLD cleavage in macrophages in vitro. Treatment of macrophages with ConA resulted in only minimal generation of the CP25 cleavage fragment without significant reduction of full‐length CYLD protein, even with prolonged treatment (Figure 4A). Similarly, increasing concentrations of ConA failed to induce substantial CYLD cleavage (Figure S5B). This marginal effect suggested potential contamination by an extrinsic factor. Since endotoxin is a common contaminant in biological preparations, we treated cells with ConA in the presence of the endotoxin inhibitor. This treatment significantly reduced ConA‐induced CYLD cleavage (Figure 4B), indicating that ConA itself does not directly trigger CYLD cleavage in macrophages. Furthermore, although ConA is a potent T cell activator [48, 49], we observed no evidence of full‐length CYLD reduction or CP25 fragment generation in T cells (Figure 4C).

Macrophages orchestrate T cell activation, polarization, and metabolism, while T cells modulate macrophage functional phenotypes through cytokines and direct contact, forming a positive feedback loop that perpetuates inflammation [50, 51, 52]. We therefore hypothesized that CYLD cleavage in macrophages requires interaction with T cells. To test this, we established a co‐culture system and observed that WT macrophages exhibited a significant decrease in full‐length CYLD, accompanied by CP25 fragment generation when co‐cultured with T cells (Figure 4D). In contrast, *Cyld^D215A/D215A^* macrophages displayed neither reduction of full‐length CYLD nor production of the CP25 fragment under identical conditions (Figure 4D), demonstrating that D215 site‐specific CYLD cleavage occurs in macrophages. To further determine the cellular specificity of this cleavage event, we co‐cultured T cells with *Cyld^−/−^* macrophages, thereby eliminating any potential contribution from macrophage CYLD (Figure 4D). Importantly, this experimental setup revealed no detectable CYLD cleavage at the D215 site in T cells (Figure 4D), providing definitive evidence that the proteolytic processing is macrophage‐specific. Taken together, these results demonstrated that the CYLD cleavage occurred exclusively in macrophages rather than in T cells within this co‐culture system.

To delineate the mechanism underlying T cell‐induced CYLD cleavage in macrophages during ConA stimulation, we systematically evaluated cell‐contact dependency through two orthogonal approaches: (i) transwell co‐culture (0.4 µm pore) to permit soluble factor exchange while preventing direct cellular interaction (Figure 4E), and (ii) administration of conditioned medium from ConA‐activated T cells to isolated macrophages (Figure 4F). Remarkably, neither paracrine signaling (transwell system) nor secreted factors (conditioned medium) could recapitulate CYLD cleavage, providing definitive evidence that this proteolytic event strictly requires direct membrane contact between T cells and macrophages.

Previous studies have shown that activated T cells express TNFα and the membrane‐bound protein FasL during interaction with macrophages or dendritic cells [53]. Furthermore, we have demonstrated that TNFα is important for inducing macrophage CYLD cleavage and EAH pathogenesis. Therefore, we measured TNFα production in our co‐culture system. While ConA stimulation alone failed to induce TNFα in either T cells or macrophages individually, significant TNFα production was observed in co‐cultures (Figure 4G), and additional analyses indicated that ConA exposure modulates the magnitude, but not the interpretation, of the TNFα readout (Figure S5C). Direct TNFα treatment induced CYLD cleavage in macrophages but not in T cells (Figure 4H–J). Moreover, when *Tnfr1^−/−^* macrophages were co‐cultured with T cells, they no longer showed ConA‐induced CYLD cleavage (Figure 4K). Consistent with this, *Tnfr1^−/−^* mice exhibited no reduction in full‐length CYLD or generation of CP25 fragments in liver tissue following ConA‐induced EAH (Figure 4L).

These results demonstrate that ConA stimulation promotes TNFα production through T cell‐macrophage interaction, which in turn induces TNFα‐dependent CYLD cleavage in macrophages.

### 2.5 CYLD Reduction by Cleavage Enhances S100A9‐Induced Chemokine Expression in Macrophages

Our findings establish that TNFα‐mediated CYLD cleavage in macrophages drives enhanced chemokine production. We further investigated whether TNFα could directly induce chemokine secretion in macrophages. Treatment with TNFα alone induced only moderate upregulation of Cxcl1/2/3 in macrophages, with *Cyld^D215A/D215A^* macrophages exhibiting marginally reduced Cxcl1 levels compared to WT (Figure S6A). These results indicate that while TNFα induces CYLD cleavage in macrophages, it cannot directly trigger robust chemokine production.

**FIGURE 5:**
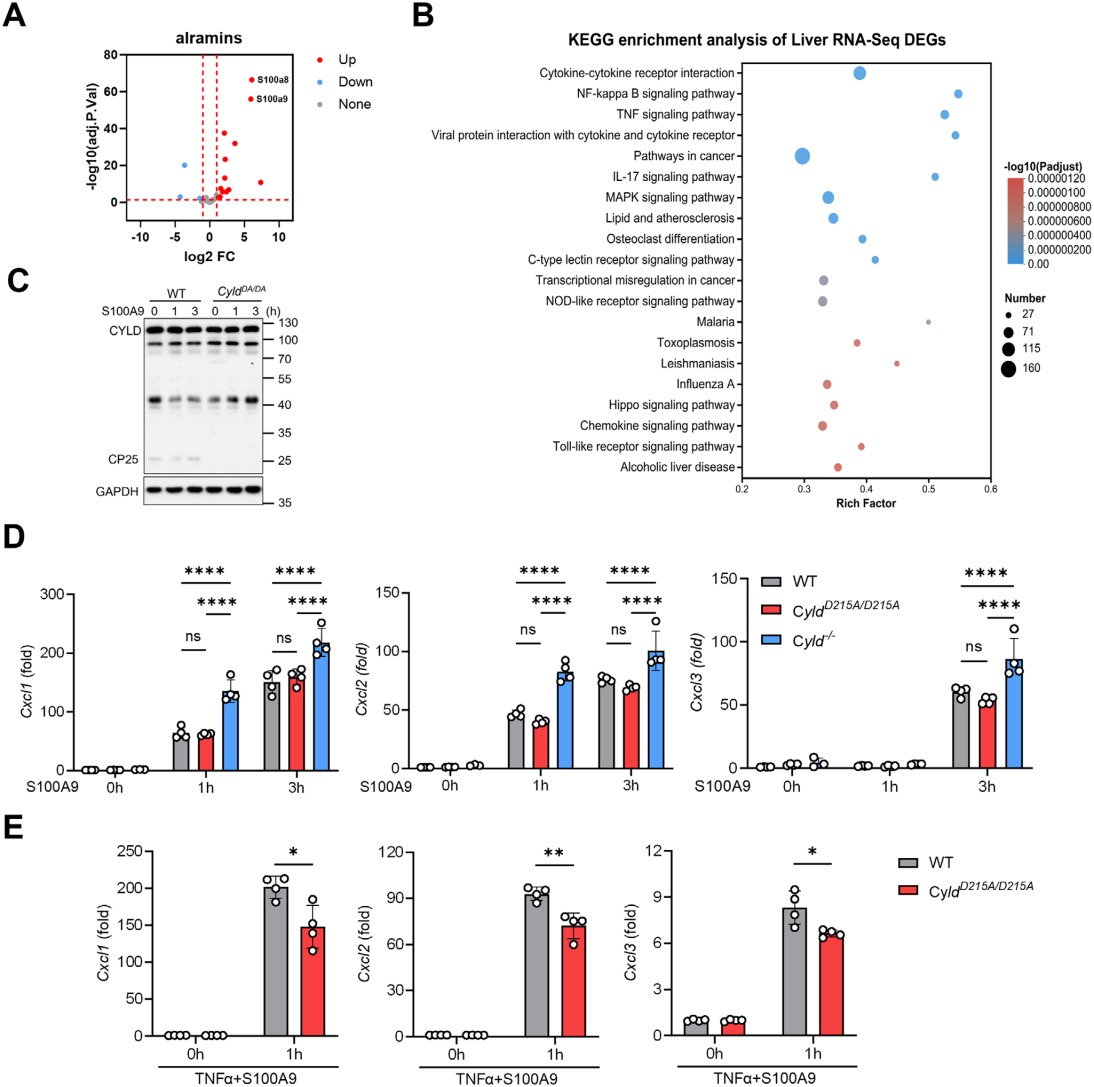
CYLD deficiency enhances S100A9‐induced chemokine expression in macrophages. (A) Volcano plot of differentially expressed alarmins in liver tissues from WT mice after intravenous ConA injection (12 mg/kg; 3 h vs. 0 h). (B) KEGG pathway enrichment analysis of differentially expressed genes (DEGs) in WT livers (3 h vs. 0 h post‐ConA). (C) Immunoblot analysis of PMs from the indicated genotypes stimulated with S100A9 (400 ng/mL) for the indicated durations. (D) Gene expression (RT‐qPCR) in genotype‐varied PMs after S100A9 (400 ng/mL) treatment for the indicated durations (n ≥ 3 biological replicates). (E) Gene expression (RT‐qPCR) in genotype‐varied PMs pretreated with mouse TNFα (20 ng/mL) for 9 h, followed by stimulation with S100A9 (400 ng/mL, 1 h) (n = 4 biological replicates). Statistical analysis for panel D was performed using two‐way ANOVA followed by Tukey's multiple comparisons test. Statistical analysis for panel E was performed using a two‐tailed unpaired Student's *t*‐test. Data are presented as mean ± SD, *p < 0.05, **p < 0.01, ***p < 0.001, ****p < 0.0001; ns, not significant.

Alarmins play crucial roles in immune regulation and leukocyte recruitment [29, 30]. Previous studies have reported that S100A8/A9 alarmins mediate neutrophil infiltration during liver injury [54], and HMGB1 deficiency alleviates hepatic damage [55]. In our EAH model, we detected marked upregulation of S100A8 and S100A9 alarmins in liver tissue (Figure 5A). These alarmins predominantly activate MAPK and NF‐κB signaling pathways [56, 57, 58], both of which showed significant enrichment in our KEGG pathway analysis of EAH liver samples (Figure 5B). When we examined the chemokine‐inducing capacity of S100A8, S100A9, and HMGB1, we found that S100A9 exhibited the most robust effect (Figure S6B). Furthermore, we found that S100A9 cannot directly induce CYLD cleavage in macrophages (Figure 5C). Building upon our established finding that cleavage‐mediated CYLD degradation in macrophages drives enhanced chemokine expression, we therefore hypothesized that intracellular CYLD levels modulate macrophage responsiveness to S100A9. To test this hypothesis, we utilized three genetically distinct macrophage models: WT, *Cyld^D215A/D215A^*, and *Cyld^−/−^*. Strikingly, *Cyld^−/−^* macrophages exhibited exaggerated responsiveness to S100A9, producing significantly more chemokines than both WT and *Cyld^D215A/D215A^* macrophages (Figure 5D). In contrast, WT and *Cyld^D215A/D215A^* macrophages showed comparable chemokine production upon S100A9 stimulation (Figure 5D), consistent with their similar CYLD protein levels. Given that TNFα triggers CYLD cleavage [37], we hypothesized that TNFα pretreatment might potentiate S100A9‐induced chemokine production. Indeed, TNFα pretreatment of WT macrophages (CYLD‐cleavable) resulted in significantly greater S100A9‐induced chemokine production compared to *Cyld^D215A/D215A^* macrophages (CYLD‐stable) (Figure 5E), establishing TNFα‐mediated CYLD degradation as a critical regulator of alarmin responsiveness.

These results reveal that the alarmin S100A9 is significantly upregulated and serves as the principal mediator of macrophage‐derived chemokine production during EAH. TNFα stimulation induces site‐specific cleavage of CYLD at D215, resulting in decreased CYLD protein abundance. This CYLD deficiency potentiates macrophage hyperresponsiveness to S100A9, driving excessive chemokine production that promotes neutrophil recruitment and amplifies hepatic inflammatory responses.

### 2.6 CYLD Inhibits Macrophage S100A9 Signaling by Deubiquitinating MEK1/2

**FIGURE 6:**
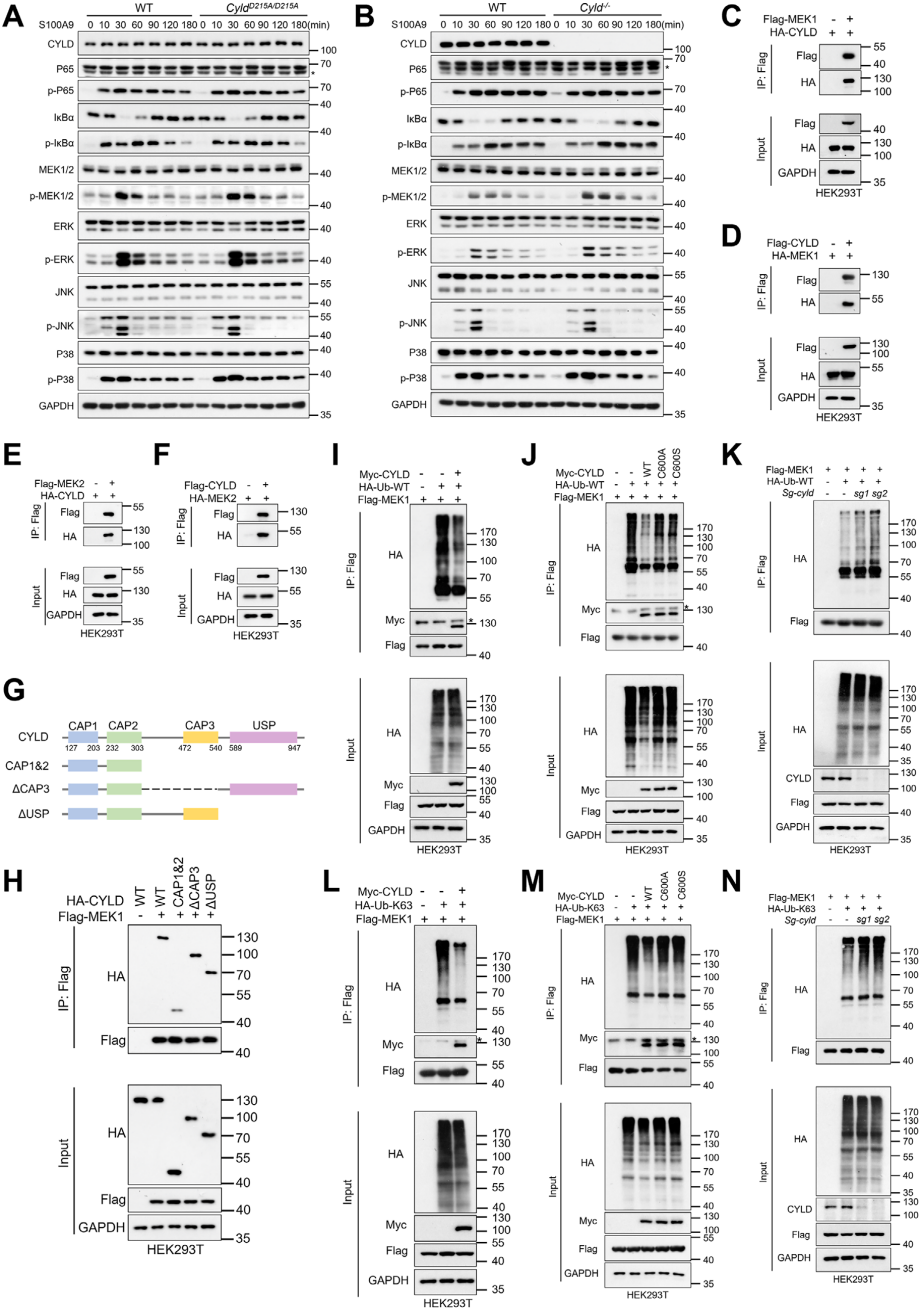
CYLD inhibits MEK1/2 activation by removing K63‐linked ubiquitin chains. (A,B) Immunoblot analysis of MAPK and NF‐κB signaling in PMs of the indicated genotypes after stimulation with S100A9 (400 ng/mL). (C–F) HEK293T cells were transfected with the indicated plasmids or corresponding empty vector for 24 h, followed by immunoprecipitation with anti‐Flag beads and immunoblot analysis. (G) Domain architecture of full‐length CYLD and truncation mutants. (H) HEK293T cells were transfected with Flag‐MEK1 and HA‐CYLD (or truncation mutants) for 24 h. Cell lysates were subjected to immunoprecipitation with anti‐Flag beads and immunoblotted with the indicated antibodies. (I,J) HEK293T cells were transfected with Flag‐MEK1, Myc‐CYLD (or catalytic mutants C600A/S), HA‐Ub‐WT, and the corresponding empty vector as indicated for 24 h. Cell lysates were subjected to immunoprecipitation with anti‐Flag beads and immunoblotted with the indicated antibodies. (K) HEK293T cells stably expressing control or *Cyld* single‐guide (sg) RNA were transfected with Flag‐MEK1, HA‐Ub‐WT, and empty vector as indicated for 24 h. Cell lysates were subjected to immunoprecipitation with anti‐Flag beads and immunoblotted with the indicated antibodies. (L,M) HEK293T cells were transfected with Flag‐MEK1, Myc‐CYLD (or catalytic mutants C600A/S), HA‐Ub‐K63, and corresponding empty vectors as indicated for 24 h. Cell lysates were subjected to immunoprecipitation with anti‐Flag beads and immunoblotted with the indicated antibodies. (N) HEK293T cells stably expressing control or *Cyld* single‐guide RNA were transfected with Flag‐MEK1, HA‐Ub‐K63, and empty vectors as indicated for 24 h. Cell lysates were subjected to immunoprecipitation with anti‐Flag beads and immunoblotted with the indicated antibodies.

To elucidate the molecular mechanism underlying enhanced macrophage sensitivity to S100A9 upon CYLD depletion, we examined NF‐κB and MAPK signaling pathways following S100A9 stimulation. Since S100A9 failed to induce CYLD cleavage, CYLD levels showed no significant difference between WT and *Cyld^D215A/D215A^* macrophages (Figure 5C). Consequently, S100A9‐induced activation of both NF‐κB and MAPK signaling pathways exhibited comparable patterns in these cells (Figure 6A). However, in *Cyld^−/−^* cells, we observed markedly upregulated phosphorylation of MEK1/2 and its downstream effector ERK1/2 within the MAPK signaling (Figure 6B). Given that MEK1/2 acts as an upstream regulatory signal, we investigated whether CYLD directly interacts with MEK1/2 proteins. Co‐immunoprecipitation assays confirmed an interaction between CYLD and MEK1/2 (Figure 6C–F), with further mapping demonstrating that amino acids 304‐955 of CYLD mediate this interaction (Figure 6G,H).

Ubiquitination generates diverse polyubiquitin chain topologies, including K6‐, K11‐, K27‐, K29‐, K33‐, K48‐, K63‐, and M1‐linked chains, which mediate distinct cellular functions. Previous studies established CYLD as a deubiquitinase that preferentially cleaves K63‐linked polyubiquitin chains from substrate proteins. Building on these findings, we hypothesized that CYLD regulates MEK1/2 signaling through K63‐linked deubiquitination. First, co‐expression of wild‐type CYLD with MEK1 in 293T cells significantly reduced MEK1 ubiquitination levels (Figure 6I). This activity was strictly dependent on CYLD's catalytic function, as mutation of critical residues (C600A/S) abolished the deubiquitination effect (Figure 6J). Genetic ablation of CYLD using CRISPR/Cas9 conversely, resulted in MEK1 hyperubiquitination (Figure 6K), confirming CYLD's physiological role in regulating MEK1 ubiquitination. Notably, the cleavage‐resistant mutant CYLD‐D215A retained the ability to bind MEK1/2 and exhibited full deubiquitination activity (Figure S7A,B). In contrast, the proteolytic CP25 fragment, generated upon cleavage of WT CYLD, neither interacted with MEK1/2 nor reduced its ubiquitination (Figure S7A,B), indicating that loss of the C‐terminal domain ablates both binding and enzymatic function.

Importantly, linkage‐specific analysis revealed that CYLD specifically removes K63‐linked polyubiquitin chains from MEK1/2 (Figure 6L). Consistent with its catalytic requirement, CYLD(C600A/S) mutants failed to reduce K63‐linked ubiquitination (Figure 6M), while CYLD deficiency increased MEK1 K63‐ubiquitination (Figure 6N). Taken together, these results demonstrate that CYLD suppresses macrophage S100A9 signaling by regulating the MEK1/2‐ERK pathway and establishes CYLD as a direct regulator of MEK1/2 via K63‐linked deubiquitination.

### 2.7 TRIM25 Cooperates With CYLD to Regulate MEK1/2 Ubiquitination

Protein ubiquitination is precisely controlled through the coordinated actions of E3 ubiquitin ligases and deubiquitinases [59]. Having demonstrated that CYLD removes K63‐linked ubiquitin chains from MEK1/2, we next sought to identify the E3 ubiquitin ligase that mediates MEK1 K63‐ubiquitination. The human genome encodes over 600 ubiquitin ligases, among which the tripartite motif (TRIM) family includes over 70 members [60, 61]. These TRIM proteins are characterized by a conserved N‐terminal TRIM/RBCC motif that invariably contains: (i) a RING finger domain conferring E3 ligase activity, (ii) one or two B‐box domains involved in protein–protein interactions, and (iii) a coiled‐coil region mediating oligomerization [60, 61]. TRIM family members play important roles in various physiological and pathological processes [60, 61]. Through mass spectrometry analysis, we identified six TRIM family members interacting with MEK1: TRIM11, TRIM21, TRIM25, TRIM32, TRIM33, and TRIM65 (Figure S8A). Notably, TRIM21, TRIM25, TRIM32, TRIM33, and TRIM65 were previously reported to mediate K63‐linked ubiquitination [62, 63, 64, 65, 66]. Among these, TRIM32 and TRIM65 showed very low peptide coverage (2 and 1 unique peptides, respectively) (Figure S8B), suggesting a weaker association. Database queries (Human Protein Atlas) further indicated that TRIM33 is primarily expressed in basophils, while both TRIM21 and TRIM25 are highly expressed in neutrophils and macrophages—a profile more relevant to our macrophage‐focused study. Preliminary validation confirmed that TRIM21 could ubiquitinate MEK1/2, though with lower efficiency than TRIM25 (Figure S8C,D). Given its established roles in inflammatory pathogenesis [67] and its stronger activity observed here, we hypothesized that TRIM25 might be a key regulator of MEK1/2 ubiquitination.

**FIGURE 7:**
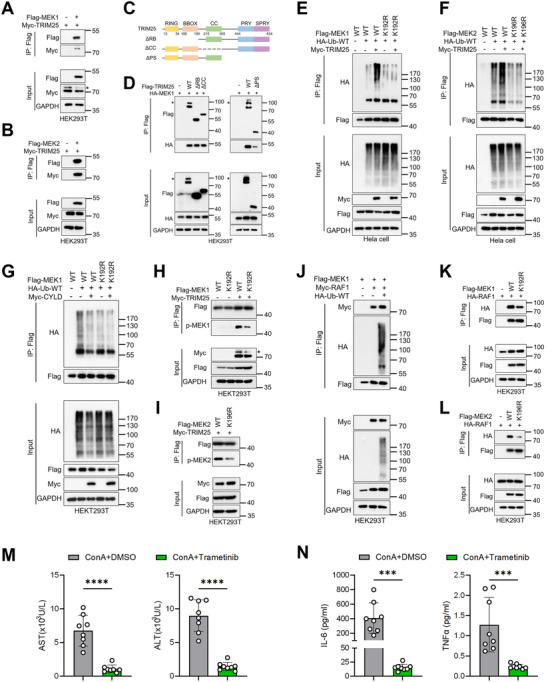
TRIM25 cooperates with CYLD to regulate MEK1/2 ubiquitination and activation. (A,B) HEK293T cells were transfected with Myc‐TRIM25 and Flag‐MEK1 (or Flag‐MEK2) with the indicated combinations for 24 h. Cell lysates were subjected to immunoprecipitation with anti‐Flag beads and immunoblotted with the indicated antibodies. (C) Domain architecture of full‐length TRIM25 and truncation mutants. (D) HEK293T cells were transfected with HA‐MEK1 and Flag‐TRIM25 (or truncation mutants) for 24 h. Cell lysates were subjected to immunoprecipitation with anti‐Flag beads and immunoblotted with the indicated antibodies. (E,F) HeLa cells were transfected with HA‐Ub‐WT, Myc‐TRIM25, and Flag‐MEK1/2 (or mutants) with the indicated combinations for 24 h. Cell lysates were subjected to immunoprecipitation with anti‐Flag beads and immunoblotted with the indicated antibodies. (G) HEK293T cells were transfected with HA‐Ub‐WT, Myc‐CYLD, and Flag‐MEK1 (or mutants) with the indicated combinations for 24 h. Cell lysates were subjected to immunoprecipitation with anti‐Flag beads and immunoblotted with the indicated antibodies. (H,I) HEK293T cells were transfected with Myc‐TRIM25 and Flag‐MEK1/2 (or mutants) with the indicated combinations for 24 h. Cell lysates were subjected to immunoprecipitation with anti‐Flag beads and immunoblotted with the indicated antibodies. (J) HEK293T cells were transfected with Flag‐MEK1, Myc‐RAF1, and HA‐Ub‐WT with the indicated combinations for 24 h. Cell lysates were subjected to immunoprecipitation with anti‐Flag beads and immunoblotted with the indicated antibodies. (K,L) HEK293T cells were transfected with HA‐RAF1 and Flag‐MEK1/2 (or mutants) with the indicated combinations for 24 h. Cell lysates were subjected to immunoprecipitation with anti‐Flag beads and immunoblotted with the indicated antibodies. (M,N) WT mice were pretreated with trametinib (5 mg/kg, i.p.) or vehicle 2 h before ConA challenge (12 mg/kg, i.v.). Serum levels of ALT, AST, IL‐6, and TNFα were measured at 12 h post‐injection (n = 8 mice per group). (M) AST and ALT. (N) IL‐6 and TNFα. Statistical analysis for panels M and N was performed using a two‐tailed unpaired Student's *t*‐test. Data are presented as mean ± SD, *p < 0.05, **p < 0.01, ***p < 0.001, ****p < 0.0001.

Co‐immunoprecipitation assays confirmed the interaction between MEK1/2 and TRIM25 (Figure 7A,B). To delineate the structural determinants of the TRIM25‐MEK1/2 interaction, we constructed a series of TRIM25 truncation mutants (Figure 7C). We found that the interaction between TRIM25^ΔPS^ and MEK1 was significantly weakened, while TRIM25^ΔRB^ and TRIM25^ΔCC^ mutations had no effect on the interaction (Figure 7D). This indicates that the PRY‐SPRY domain of TRIM25 participates in its interaction with MEK1/2. We further investigated TRIM25‐mediated ubiquitination of MEK1/2. In 293T cells, co‐transfection of TRIM25 increased K63 ubiquitination of MEK1 in a dose‐dependent manner (Figure S8D). TRIM25^ΔRB^ failed to ubiquitinate MEK1 (Figure S8E,F), demonstrating that the RB domain is essential for E3 ligase activity of TRIM25 toward MEK1/2.

To identify specific ubiquitination sites on MEK1, we employed the GPS‐Uber prediction tool to computationally map potential ubiquitination sites (Figure S8G). Subsequent mutagenesis and functional validation identified K192 as a ubiquitination site on MEK1, where the MEK1^K192R^ mutant showed markedly reduced ubiquitination levels both upon TRIM25 co‐expression (Figure 7E; Figure S8H) and under basal conditions (Figure S8I). Sequence alignment showed conserved lysine residues between MEK1‐K192 and MEK2‐K196 (Figure S8J). A MEK2 mutation in which K196 was replaced with Arg(K196R) could not be ubiquitinated by TRIM25 (Figure 7F). Thus, TRIM25 ubiquitinates MEK2 mainly at the K196 residue. Notably, while CYLD reduced ubiquitination of MEK1, it failed to deubiquitinate the MEK1^K192R^ mutant (Figure 7G), indicating that CYLD specifically removes K63‐linked polyubiquitin chains from K192 of MEK1. These data establish that TRIM25 catalyzes K63‐linked ubiquitination at MEK1‐K192/MEK2‐K196, while CYLD reverses this modification, thereby identifying a balanced ubiquitination‐editing system that orchestrates MEK1/2 ubiquitination states.

To investigate how ubiquitination regulates MEK1/2 activation, we first examined MEK1/2 phosphorylation following TRIM25 overexpression. Strikingly, TRIM25 potently enhanced MEK1/2 activation, whereas both the MEK1^K192R^ and MEK2^K196R^ mutants exhibited significantly reduced phosphorylation (Figure 7H,I; Figure S8K), suggesting that TRIM25 promotes MEK1/2 activation through K63‐linked ubiquitination. Given the established requirement of RAF kinases for MEK1/2 activation [68], we sought to determine whether MEK1/2 ubiquitination regulates its activation by modulating the interaction with RAF1. Remarkably, MEK1 co‐expressed with ubiquitin exhibited significantly enhanced binding to RAF1 compared to non‐ubiquitinated MEK1. (Figure 7J). Conversely, both MEK1^K192R^ and MEK2^K196R^ mutants showed impaired interaction with RAF1 (Figure 7K,L), demonstrating that ubiquitination of MEKs increases their interaction with RAF1. These results demonstrate that TRIM25‐mediated K63 ubiquitination of MEK1/2 enhances their interaction with RAF1, thereby facilitating subsequent activation.

Our previous findings demonstrated significant upregulation of the MAPK signaling pathway in EAH, with MEK1/2 hyperactivation representing a critical pathogenic mechanism. To further investigate this, we pre‐treated mice with the MEK1/2 inhibitor trametinib (5 mg/kg, a dose previously shown to be well tolerated in mice [69]) 2 h before ConA injection. The trametinib‐treated group displayed markedly reduced the degree of liver necrosis compared to DMSO controls (Figure 7M; Figure S9A,B). Furthermore, MEK1/2 inhibition significantly suppressed the production of proinflammatory cytokines and chemokines (Figure 7N; Figure S9C). These findings demonstrate that pharmacological blockade of MEK1/2 effectively mitigates inflammatory responses and confers protection against EAH development. Collectively, these results identify MEK1/2 as a promising therapeutic target for AIH.

## 3 Discussion

This study elucidates a previously unappreciated role for CYLD cleavage in EAH, which results from proteolytic processing at the D215 site and leads to loss of the full‐length protein. We further unveil a previously unrecognized role of the deubiquitinase CYLD in suppressing EAH pathogenesis through direct regulation of the MAPK signaling cascade. We provide the first evidence that CYLD physically interacts with MEK1/2 and selectively removes K63‐linked ubiquitin chains from the K192/K196 residues, thereby inhibiting MEK1/2 activation and downstream MAPK signaling. This mechanistic insight is further strengthened by our observation that pharmacological inhibition of MEK1/2 with Trametinib significantly attenuates liver injury and inflammation in EAH models. Given that MAPK hyperactivation critically contributes to inflammatory pathogenesis [70], our findings establish CYLD as a master negative regulator of this pathway in AIH. Furthermore, we demonstrated that the deubiquitinase CYLD cooperates with the E3 ubiquitin ligase TRIM25 to reciprocally regulate MEK1/2 ubiquitination levels. Meanwhile, mass spectrometry analysis identified multiple additional ubiquitin‐modifying enzymes potentially interacting with MEK1 (Figure S8A), suggesting the existence of an intricate regulatory network that extends beyond this core mechanism. The precise mechanistic relationships between these newly identified proteins and MEK require further investigation.

Our data implicate alarmins, particularly S100A9, as critical amplifiers of hepatic inflammation in AIH. We observed marked upregulation of S100A8/A9 in EAH livers, where S100A9 acts as a potent chemotactic agent that activates macrophage MAPK signaling, leading to excessive chemokine secretion. This aligns with reports linking S100A8/A9 to neutrophil recruitment in acute and chronic liver injury [54]. Crucially, CYLD counteracts this process by suppressing MAPK activation, thereby blunting the pro‐inflammatory response to S100A9. The concurrent upregulation of multiple alarmins in EAH suggests their potential synergy in disease progression, a paradigm underexplored in AIH but critical for understanding its unique immunopathology.

TNFα plays a pivotal role in AIH pathogenesis, and TNFα blockers (e.g., infliximab) have been used as rescue therapy for treatment‐refractory cases in clinical practice [1, 5]. Building on reports that TNFα activates Caspase8 to cleave CYLD at D215, leading to its proteolytic degradation [37], we now establish the functional consequence of this cleavage event in AIH. Bone marrow transplantation and macrophage‐specific CYLD‐D215A knock‐in mice revealed that preventing CYLD cleavage in macrophages alone suffices to ameliorate EAH. Mechanistically, TNFα‐induced CYLD degradation hypersensitizes macrophages to alarmins (e.g., S100A9), permitting unrestrained MAPK activation and chemokine‐driven inflammation. This discovery provides a new perspective for understanding the role of TNFα in inflammatory diseases.

**FIGURE 8:**
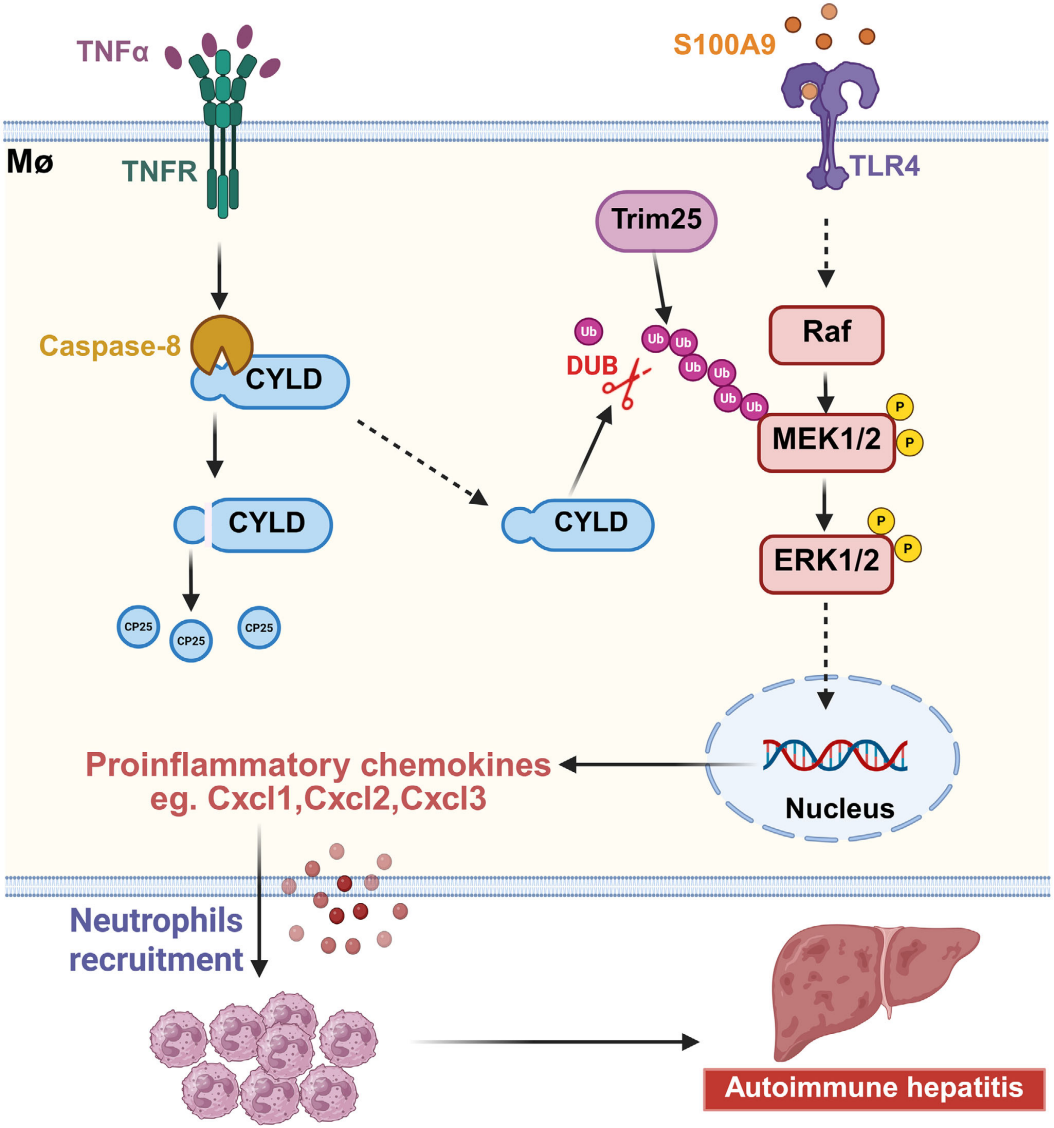
This schematic illustrates a central mechanism driving autoimmune hepatitis progression, in which the stability of the deubiquitinase CYLD in macrophages acts as a critical regulatory checkpoint. TNFα signaling activates Caspase‐8, leading to proteolytic cleavage of CYLD at Asp215 and loss of functional full‐length CYLD. CYLD inactivation relieves its negative regulation of the MEK1/2–ERK1/2 signaling pathway, thereby sensitizing macrophages to alarmin stimulation, particularly S100A9. Sustained MAPK hyperactivation results in excessive chemokine production, which promotes neutrophil recruitment and infiltration into the liver, ultimately exacerbating hepatic inflammation and driving autoimmune hepatitis progression.

Our integrated model (Figure 8) proposes that TNFα‐driven CYLD degradation and S100A9‐mediated MAPK hyperactivation constitute cooperative pathways driving AIH pathogenesis. This model suggests three complementary therapeutic strategies: (1) Dual‐pathway blockade combining TNFα inhibitors (e.g., infliximab) with S100A9 antagonists (e.g., paquinimod, under clinical evaluation for inflammatory disorders); (2) CYLD stabilization via Caspase8 inhibitors (e.g., emricasan) or CYLD‐enhancing agents; and (3) Precision modulation of the MAPK network using MEK1/2 inhibitors (trametinib) or antagonists targeting ubiquitin‐regulating enzymes (e.g., TRIM25). In this study, the therapeutic potential of MEK inhibition was demonstrated by a single, well‐tolerated dose of trametinib (5 mg/kg [69]). However, given the systemic toxicities associated with prolonged MEK inhibitor treatment [71, 72], clinical translation will likely require improved cell‐type and/or tissue specificity. Future studies should therefore focus on developing macrophage‐targeted and/or liver‐enriched delivery strategies to preferentially restrict MEK pathway inhibition to hepatic macrophages. In addition, further investigation is warranted to assess the clinical relevance of CYLD cleavage and S100A9 levels in serum and liver biopsy samples from patients with AIH.

In summary, our study reveals a pivotal mechanistic link in AIH pathogenesis: TNFα‐triggered proteolytic cleavage of CYLD in macrophages diminishes its regulatory function, thereby permitting S100A9‐mediated hyperactivation of MAPK signaling. This cascade culminates in excessive chemokine production and intensified hepatic inflammatory recruitment. These insights substantially advance our pathophysiological understanding of AIH while establishing a molecular framework for targeted therapeutic development.

## 4 Experimental Section

### 4.1 Mice

The mouse *Cyld* D215A mutation construct corresponded to the following genomic position (NC_000074.7) chr13: 89423506‐89478574. Genotyping was performed using primers 5′‐ATTATGATTTGAAACGATGAGG‐3′ and 5′‐CTGGACCAAAGATAAACTGAA‐3′, which yielded a 627‐bp amplicon for sequencing. *Cyld ^loxp‐WT‐stop‐loxp‐D215A^* conditional knock‐in mice (designated *D215A^f/f^*) were generated by homologous recombination in embryonic stem cells. The targeting cassette contained a *loxP‐*flanked wild‐ type *cyld* cDNA (exons 3 to18) followed by a stop codon and a *loxP‐*flanked mutant *Cyld* cDNA carrying the D215A mutation in exon 3, which replaced the endogenous exon 3. These mice were genotyped with primers 5′‐ TCAAGGTTTCACGGATGGGG‐3′ and 5′‐ GCACATGCATTCTGTGTCTGC‐3′, yielding a 467‐bp amplicon for sequencing. In the absence of Cre recombinase, these mice express WT CYLD protein. *D215A^f/f^* mice were crossed with *LysM‐cre* and *Cd4‐cre* mice to generate macrophage‐ and T cell‐specific CYLD‐D215A mutants, respectively. The *LysM‐Cre* mice and *Cd4‐Cre* mice were purchased from Shanghai Model Organisms Center. *Cyld^−/−^* mic were generated using CRISPR‐Cas9 technology with two single‐guide RNAs (sgRNAs): sgRNA1, 5′‐ TACTGTCCTATACTCCCTTT ‐3′; sgRNA 2, 5′‐ GGGACTTACAGCGAGTTCAT ‐3′. Genotyping was performed using primers 5′‐GGGACTTACAGCGAGTTCAT‐3′ and 5′‐ ATAGATCAGTGGTAGAGGGT‐3′, which amplified a 778‐bp fragment from the WT allele and a 349‐bp fragment from the knockout allele. *Tnfr1^−/−^* mice were described previously [73].

All mice were backcrossed for more than eight generations onto a C57BL/6 background and housed under specific pathogen‐free (SPF) conditions at the Shanghai Institute of Nutrition and Health, Chinese Academy of Sciences. All animal experiments were approved by the Institutional Animal Care and Use Committee (IACUC) of the Shanghai Institute of Nutrition and Health, Chinese Academy of Sciences (Approval No. SINH‐2025‐ZHB‐1), and were performed in accordance with institutional guidelines.

### 4.2 ConA‐Induced Autoimmune Hepatitis Model

Concanavalin A (ConA; Sigma, C2010) was dissolved in sterile phosphate‐buffered saline (PBS) to prepare a 2 mg/mL stock solution. Autoimmune hepatitis was induced in 6‐ to 8‐week‐old mice via intravenous (i.v.) injection of ConA (12 mg/kg) into the tail vein. Tissues and sera were collected at the indicated time points post‐injection. For survival studies, mice received a lethal dose of ConA (25 mg/kg, i.v.), and survival was monitored and recorded.

### 4.3 Bone Marrow Transfer Experiment

Recipient mice were lethally irradiated with a total dose of 11 Gy (split into two doses of 5.5 Gy separated by 4 h) using an X‐ray irradiator. Bone marrow (BM) cells were isolated from donor mice, and erythrocytes were lysed. A total of 5 × 10^6^ erythrocyte‐depleted BM cells were injected intravenously via the tail vein into each irradiated recipient. Recipient mice received water supplemented with antibiotics (2 mg/mL penicillin and 1.5 mg/mL streptomycin) for 2 weeks and were allowed to reconstitute for 60 days before subsequent experiments.

### 4.4 Trametinib Administration in ConA‐Induced Autoimmune Hepatitis Model

Trametinib (MedChemExpress (MCE), HY‐10999) was dissolved in dimethyl sulfoxide (DMSO) to a 20 mg/mL stock solution, aliquoted, and stored at −80°C. Prior to injection, the stock was diluted in vehicle (2.5% DMSO, 20% PEG 300, 2.5% Tween‐80, 75% PBS) to a working concentration of 0.5 mg/mL. Mice received Trametinib (5 mg/kg body weight) or an equivalent volume of vehicle control via intraperitoneal injection 2 h prior to ConA challenge.

### 4.5 Serum Transaminase and Cytokine Quantification

Blood samples were incubated at 4°C for 12–16 h to clot. Serum was obtained by centrifugation at 2000 × g for 15 min at 4°C. Alanine aminotransferase (ALT) and aspartate aminotransferase (AST) activities were measured using commercial kits (Shanghai Shensuo UNF). Serum levels of TNFα and IL‐6 were quantified using ELISA kits (eBioscience, 88‐7324‐88 and 88‐7064‐88, respectively) according to the manufacturer's protocols.

### 4.6 Flow Cytometric Analysis

Following blood exsanguination, liver and spleen tissues were harvested and mechanically homogenized to obtain single‐cell suspensions by passage through a 70‐µm cell strainer. The suspension was centrifuged at 50 × g for 5 min to pellet the hepatocytes. The supernatant, enriched in liver leukocytes, was centrifuged at 500 × g for 10 min at 4°C. The pellet was resuspended in 10 mL 40% Percoll (GE Healthcare, 17‐0891‐02), followed by centrifugation at 800 × g for 25 min at 4°C. The resulting pellet was resuspended in 2 mL of ACK lysing buffer for 3 min, washed by centrifugation, and then resuspended in staining buffer (PBS supplemented with 0.5% BSA) for cell counting and subsequent flow cytometry analysis.

For immunophenotyping, aliquots of cells were stained with antibodies of interest in the dark for 30 min at 4°C. The following antibodies were used: CD45‐FITC (BioLegend, 103 108), CD11b‐APC (eBioscience, 17‐0112‐82), Ly6G‐PE‐CY7 (BioLegend, 127 617), F4/80‐BV421(BioLegend, 123 132), Ly6C‐PerCP (BD Biosciences, 560 525), CD45‐BV421 (BioLegend,103 134), CD3‐FITC (BD Biosciences, 553 061), NK1.1‐PE (BioLegend, 108 707), CD19‐PE‐CY7 (Invitrogen, 25‐0193‐82). After staining, cells were washed twice with staining buffer and analyzed using a CytoFLEX S flow cytometer (Beckman Coulter). Data were processed using FlowJo and CytoExpert software.

### 4.7 Cell Culture and Treatment

All cells were cultured at 37°C in a 5% CO_2_ incubator. PMs were isolated from 8‐week‐old C57BL/6J mice. Mice received an intraperitoneal injection of 3 mL sterile 3% thioglycollate medium (BD Biosciences, 211 716). After 72 h, peritoneal exudate cells were harvested by lavage with ice‐cold PBS and centrifuged at 500 × g for 3 min. Cells were resuspended in RPMI 1640 medium (Gibco) supplemented with 10% heat‐inactivated fetal bovine serum (FBS; HyClone, SH30084.03), 1% penicillin and streptomycin (ThermoFisher, 15140122). PMs were plated in 6‐well plates at 2 × 10^6^ cells/well and cultured overnight (16–18 h) in complete RPMI 1640 medium. Cells were then treated with recombinant proteins at specified time points: TNFα (20 ng/mL; R&D Systems, 410‐MT), S100A8 (400 ng/mL; MCE, HY‐P73671), S100A9 (400 ng/mL; MCE, HY‐P74583), or HMGB1 (400 ng/mL; MCE, HY‐73104A). Polymyxin B Sulfate (25 µg/mL; MCE, HY‐A0248).

Splenic CD3^+^ T cells were isolated following manuscripts (Stemcell, 19851) and cultured with complete RPMI 1640 medium. HEK293T cells were cultured in Dulbecco's Modified Eagle's Medium (SH30243.LS, Hyclone) supplemented with 10% heat‐inactivated fetal bovine serum, 1% penicillin/streptomycin.

### 4.8 Western Blotting and Immunoprecipitation

Cell pellets and tissues were lysed in ice‐cold RIPA lysis buffer (50 mm Tris‐HCl pH8.0, 150 mm NaCl, 1% Triton X‐100, 0.1% SDS, 1 mm EDTA, 50 mm NaF, 0.2% sodium deoxycholate), supplemented with protease inhibitor: 2 mm PMSF, 5 mm NEM, 1× proteinase inhibitor cocktail (Roche, 5056489001). Lysates were centrifuged at 12 000 × g for 30 min at 4°C. Supernatants were collected and protein concentrations determined using a BCA assay kit (Beyotime, P0010S). Samples were mixed with 5 × SDS loading sample buffer denatured at 100°C for 10 min, and stored at −80°C. The samples were analyzed using SDS‐PAGE and immunoblotting.

For immunoprecipitation assay, cells were lysed in ice‐cold NP‐40 buffer (30 mm Tris ‐HCl pH7.5, 0.5% NP‐40, 10% glycerol, 120 mm NaCl, 50 mm NaF, 10 mm β‐glycerophosphate, 5 mm sodium pyrophosphate, 2 mm KCl, 1 mm EDTA), supplemented with protease inhibitor: 2 mm PMSF, 5 mm NEM, 1× complete proteinase inhibitor cocktail. Lysates were centrifuged at 12 000 × g for 30 min at 4°C. A 10% aliquot of supernatant was mixed with 5 × SDS loading sample buffer as an input control and denatured at 100°C for 10 min. The remaining supernatant was incubated with anti‐FLAG M2 affinity gel (Sigma, A2220) overnight at 4°C with rotation. The immunocomplexes were washed five times with cold IP wash buffer (100 mm Tris‐HCl pH 8, 0.15 m NaCl, 5 mm EDTA, 0.05% NP‐40). Bound proteins were eluted with 2 × SDS loading sample buffer by boiling at 100°C for 10 min. The samples were analyzed using SDS‐PAGE and immunoblotting.

The following antibodies were used for western blotting and immunoprecipitation experiments: CYLD (1:1000, Cell Signaling Technology, 8462), GAPDH (1:20 000, Sigma, G9545), P65 (1:1000, Cell Signaling Technology, 8242), p‐P65 (1:1000, Cell Signaling Technology, 3033), IκBα (1:2000, Cell Signaling Technology, 9242), p‐IκBα (1:1000, Cell Signaling Technology, 9246), MEK1/2 (1:1000, Cell Signaling Technology, 9122), p‐MEK1/2 (1:1000, Cell Signaling Technology, 9121), ERK1/2 (1:1000, Cell Signaling Technology, 9102), p‐ERK1/2 (1:1000, Cell Signaling Technology, 9101), P38 (1:1000, Cell Signaling Technology, 9228), p‐P38 (1:1000, Cell Signaling Technology, 9211), JNK (1:1000, Cell Signaling Technology, 9252), p‐JNK (1:1000, Cell Signaling Technology, 9251), HA‐Tag (1:3000, Cell Signaling Technology, 3724), Myc‐Tag (1:2000, Cell Signaling Technology, 2276), Flag‐Tag (1:20 000, Sigma, A8592‐1MG), K63‐linkage specific polyubiquitin (1:1000, Cell Signaling Technology, 5621).

### 4.9 Plasmids

Murine *Cyld*, *Mek1*, *Mek2*, and *Trim25* cDNAs were cloned into pcDNA3.0‐Flag, pcDNA3.1–3 × HA, or pcDNA3.0‐Myc vectors to generate tagged expression constructs. Truncation mutants of HA‐CYLD (CAP1&2(1–303), ΔCAP3(Δ304–540), ΔUSP(Δ541–955)) and Flag‐TRIM25 (ΔRB(Δ1–190), ΔCC(Δ191–305), ΔPS(Δ306–634)) were generated from the respective full‐length templates. Point mutations in MEK1 (K36R, K57&59R, K70R, K104R, K156&157R, K168R, K175R, K192R, K205R, K344R, and K353R), MEK2 (K196R), and CYLD (C600A, C600S) were introduced by site‐directed mutagenesis. All constructs were verified by sequencing. Plasmids encoding HA‐tagged wild‐type and mutant (K63) ubiquitin were gifts from Dr. Hao Ying (Shanghai Institute of Nutrition and Health, Chinese Academy of Sciences).

### 4.10 CRISPR‐Cas9‐Mediated Cyld Knockout

For *Cyld* depletion, sgRNAs were designed: sgRNA1 targeting mouse *Cyld* (5'‐AAAGCTCCTTAAAGTACCGA‐3), sgRNA2 targeting mouse *Cyld* (5'‐GTGGTCAAGGTTTCACTGAC‐3'). Oligonucleotide duplexes were generated by annealing complementary primers: sgRNA1 (forward: 5'‐AAAGCTCCTTAAAGTACCGA‐3'; reverse: 5'‐TCGGTACTTTAAGGAGCTTT‐3'), sgRNA2 (forward: 5'‐GTGGTCAAGGTTTCACTGAC‐3'; reverse: 5'‐GTCAGTGAAACCTTGACCAC‐3'). Duplexes were individually ligated into BsmBI‐linearized lentiCRISPR v2 (Addgene, 52 961) using T4 DNA ligase (Thermo, EL0014). HEK293T cells were infected with lentiCRISPR v2‐sgCyld vectors and selected with 3 µg/mL puromycin for 7 days.

### 4.11 Cell Transfection

HEK293T cells or Hela cells in 6‐well plates were transfected with Lipofectamine 2000 (Invitrogen, 11 668 019) following manufacturer guidelines: 1 µg plasmid DNA was diluted in 100 µL Opti‐MEM I Reduced Serum Medium (Gibco, 31 985 070), mixed with 100 µL Opti‐MEM containing 2 µL Lipofectamine 2000, incubated for 20 min to form complexes, then added dropwise to cells.

### 4.12 RNA‐Seq and RT‐qPCR

Total RNAs were extracted using TRIzol Reagent (Takara, 9108) from WT untreated and treated with ConA (12 mg/kg, i.v.) for 3 h mice liver tissues. RNA samples were sequenced by Majorbio Tech Solutions. The raw transcriptomic reads were mapped to mus musculus reference (GRCm39). Based on the quantitative expression results, the differentially expressed genes were obtained between the two groups. The difference analysis software was DESeq2, and the screening threshold was |log2FC| >= 1 and padjust < 0.05.

Total RNA from cells or tissues was extracted using TRIzol Reagent. cDNA was synthesized from 1 µg RNA using the PrimeScript RT Master Mix (Takara, RR037A) according to the manufacturer's protocol. Real‐time qPCR was performed in triplicate using SYBR Premix Ex Taq (ROX Plus) (Takara, RR420A) on a QuantStudio6 Flex System. Gene expression was normalized to *Actb* (β‐actin) and calculated by the 2^−^ *^ΔΔCt^* method. Primer sequences were designed using the Primer‐Bank database (http://pga.mgh.harvard.edu/primerbank). All primer sequences are provided in Table S1.

### 4.13 Statistical Analysis

Data were presented as mean ± SD. All experiments were performed with at least three independent biological replicates, and the exact sample size (*n*) for each experiment was indicated in the figure legends. No data were excluded from the analyses. Quantitative PCR data were normalized to the indicated internal control genes and calculated using the 2^−ΔΔ*Ct*^ method. No data transformation or normalization was applied for other experiments.

Mouse survival was analyzed using the log‐rank (Mantel–Cox) test. Comparisons between two groups were performed using an unpaired two‐tailed Student's *t*‐test. For experiments involving two independent variables, statistical significance was assessed by two‐way analysis of variance (ANOVA), followed by Tukey's multiple comparisons test. A *p‐*value < 0.05 was considered statistically significant. All statistical analyses were performed using GraphPad Prism version 9.0 (GraphPad Software).

## Author Contributions

H.B.Z. and M.L. conceived the project. H.L., C.S, M.L., and H.B.Z. designed the study. H.L. performed the experiments and data analyses with assistance from M.L., C.S., J.L.L., and M.Y.X. X.X.W., L.X.W., X.M.Z., H.W.Z., Y.Y.X., and Y.Y.W. assisted with mouse breeding and analysis. H.L. and Y.L. contributed to the critical discussion. Y.L. provided essential resources and intellectual input during the experimental studies. H.L., C.S., M.L., and H.B.Z. assembled figure panels and wrote the paper. H.B.Z. supervised the project.

## Funding

This work was supported by grants from the National Science and Technology Major Project for Chronic Non‐communicable Diseases (2024ZD0531300), National Key Research and Development Program of China (2022YFA0807300), National Natural Science Foundation of China (32300630, 32270803), Shanghai Excellent Academic/Technical Leader Program (22XD1404500), Shanghai Science and Technology Commission (23141902800) and Shanghai Municipal Science and Technology Major Project.

## Conflicts of Interest

The authors declare no conflict of interest.

The data that support the findings of this study are available from the corresponding author upon reasonable request.

## Supporting information




**Supporting File**: advs74090‐sup‐0001‐SuppMat.docx.

## Data Availability

The sequencing data generated in this study are openly available in the NCBI Sequence Read Archive (SRA) under the BioProject accession number PRJNA1397907.
