# Supplementary material for: Cleavage‐Resistant CYLD Protects Against Autoimmune Hepatitis
Source: Adv Sci (Weinh). 2026 Jan 28;13(19):e13015. doi: 10.1002/advs.202513015 (PMC13045489; doi:10.1002/advs.202513015)
Supplement: Supplementary file 1 — Supporting File: advs74090‐sup‐0001‐SuppMat.docx. [file ADVS-13-e13015-s001.docx]

**Supporting Information**

**Cleavage-Resistant CYLD Protects Against Autoimmune Hepatitis**

*Han Liu^1,4^, Chen Su^3,4^, Jianling Liu^1^, Mingyan Xing^1^, Xiaoxia Wu^1^, Lingxia Wang^1^, Xiaoming Zhao^1^, Hanwen Zhang^1^, YangYang Xie^1^, YangYang Wang^1^, Hong Li^1,2^, Yu Li^1^, Ming Li^1*^, Haibing Zhang^1*^*

**
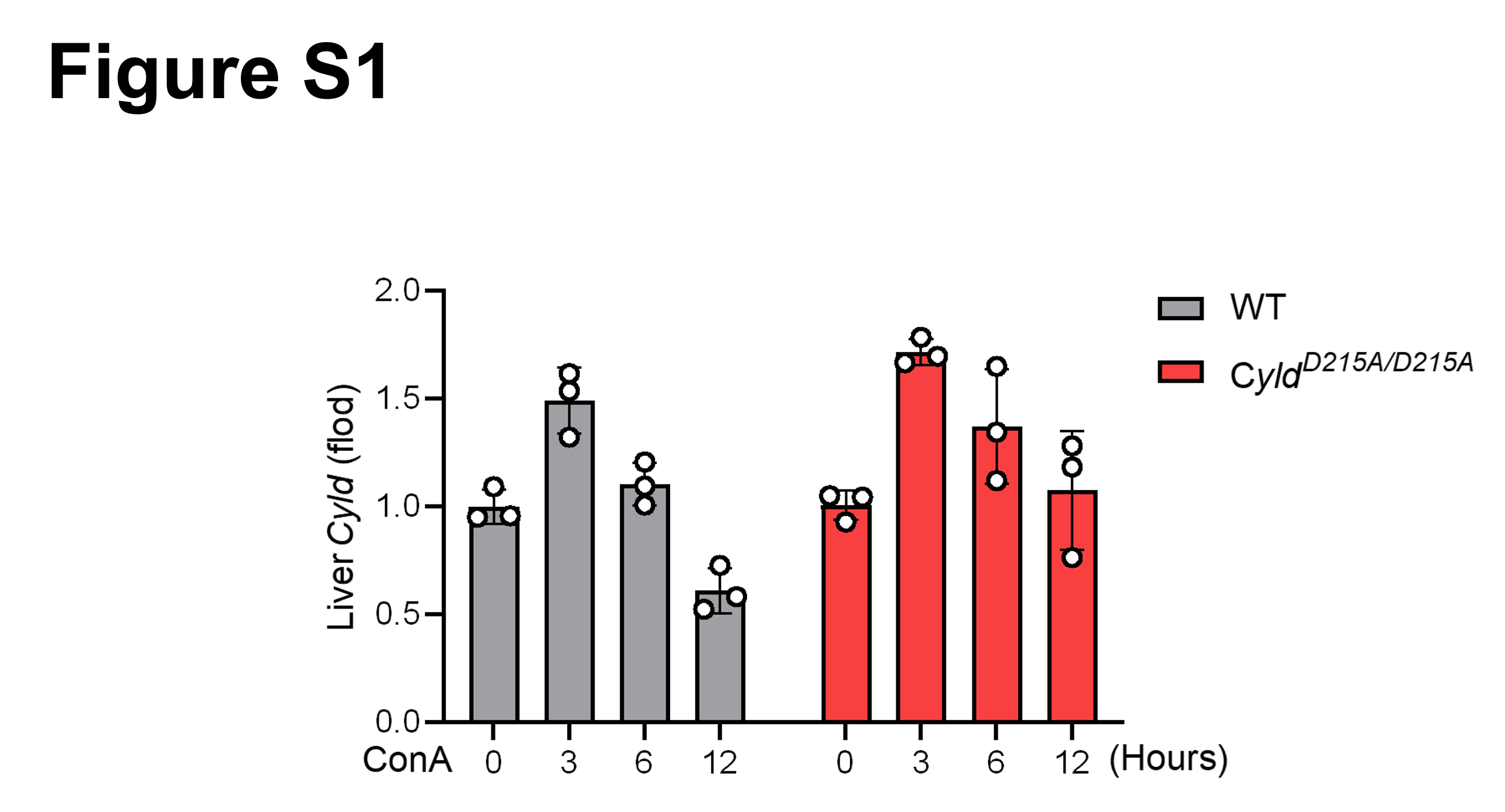
****Figure S1.** Detection of Cyld gene expression in mice. RT-qPCR analysis of *Cyld* expression in liver tissues from mice with the indicated genotypes after ConA treatment (12 mg/kg, i.v.) (n = 3 mice per group). *Cyld* expression levels were normalized to *Rn18s*.

**
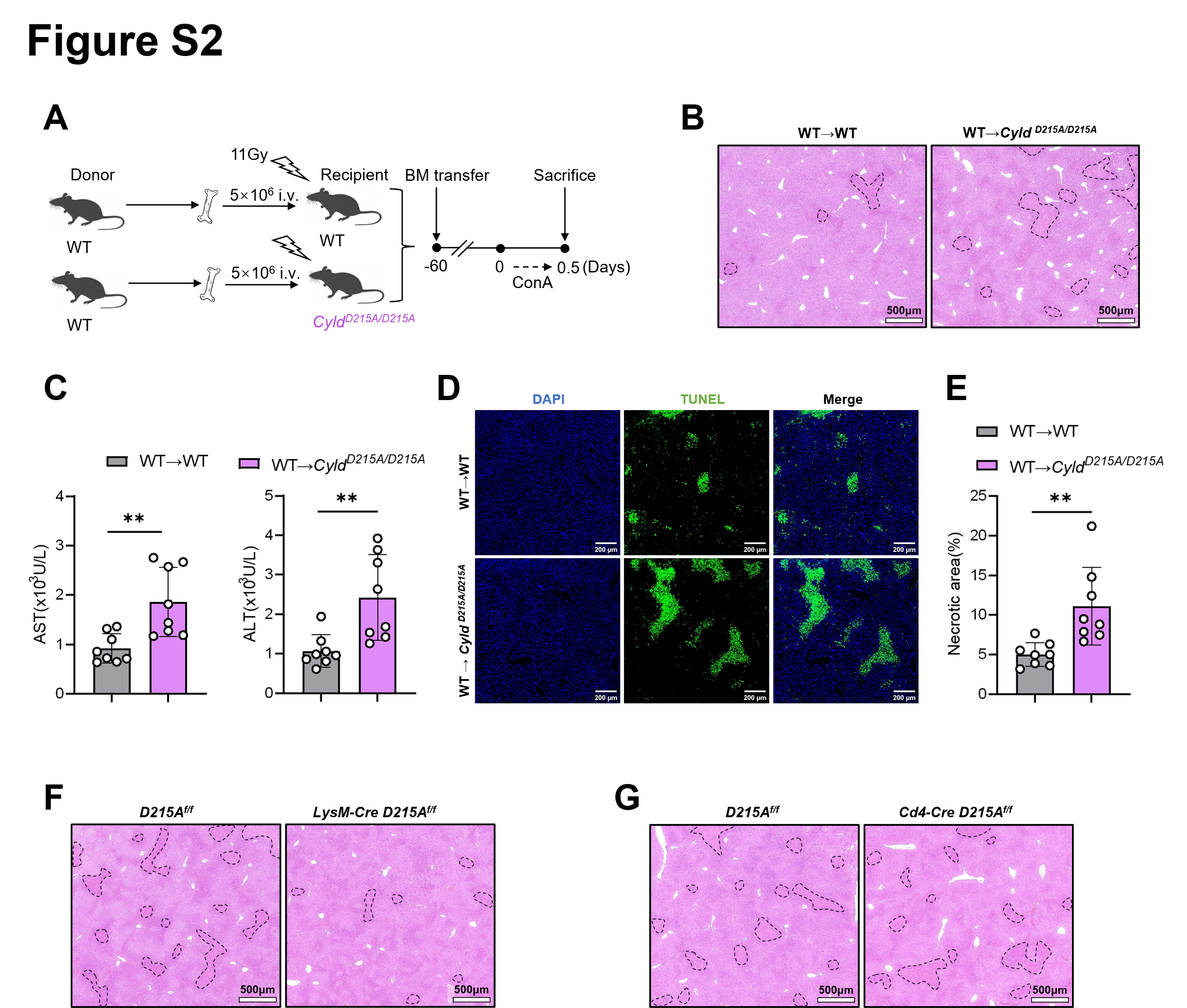
Figure S2.** Cleavage-resistant CYLD expression in non-myeloid-derived cells didn't protect against EAH. A–E) BM transfer experiments: BM cells from WT or *Cyld^D215A/D215A^* mice were transplanted into lethally irradiated (11 Gy) recipient mice. After 8 weeks of reconstitution, EAH was induced by intravenous ConA injection (12 mg/kg, i.v.; 12 h) (n = 8 mice per group). (A) Experimental design schematic. (B) Representative H&E-stained liver sections (scale bar: 500 μm). (C) Serum ALT/AST levels (U/L). (D) TUNEL staining of liver sections (scale bar: 200 μm). (E) Quantification of TUNEL-positive areas. F, G) Representative H&E-stained liver sections (scale bar: 500 μm) from mice of indicated genotypes 12 h after ConA treatment (12 mg/kg, i.v.). Statistical analysis for panels C and E was performed using a two-tailed unpaired Student's *t*-test. Data are presented as mean ± SD, **P* < 0.05, ***P* < 0.01, ****P* < 0.001, *****P* < 0.0001.

**
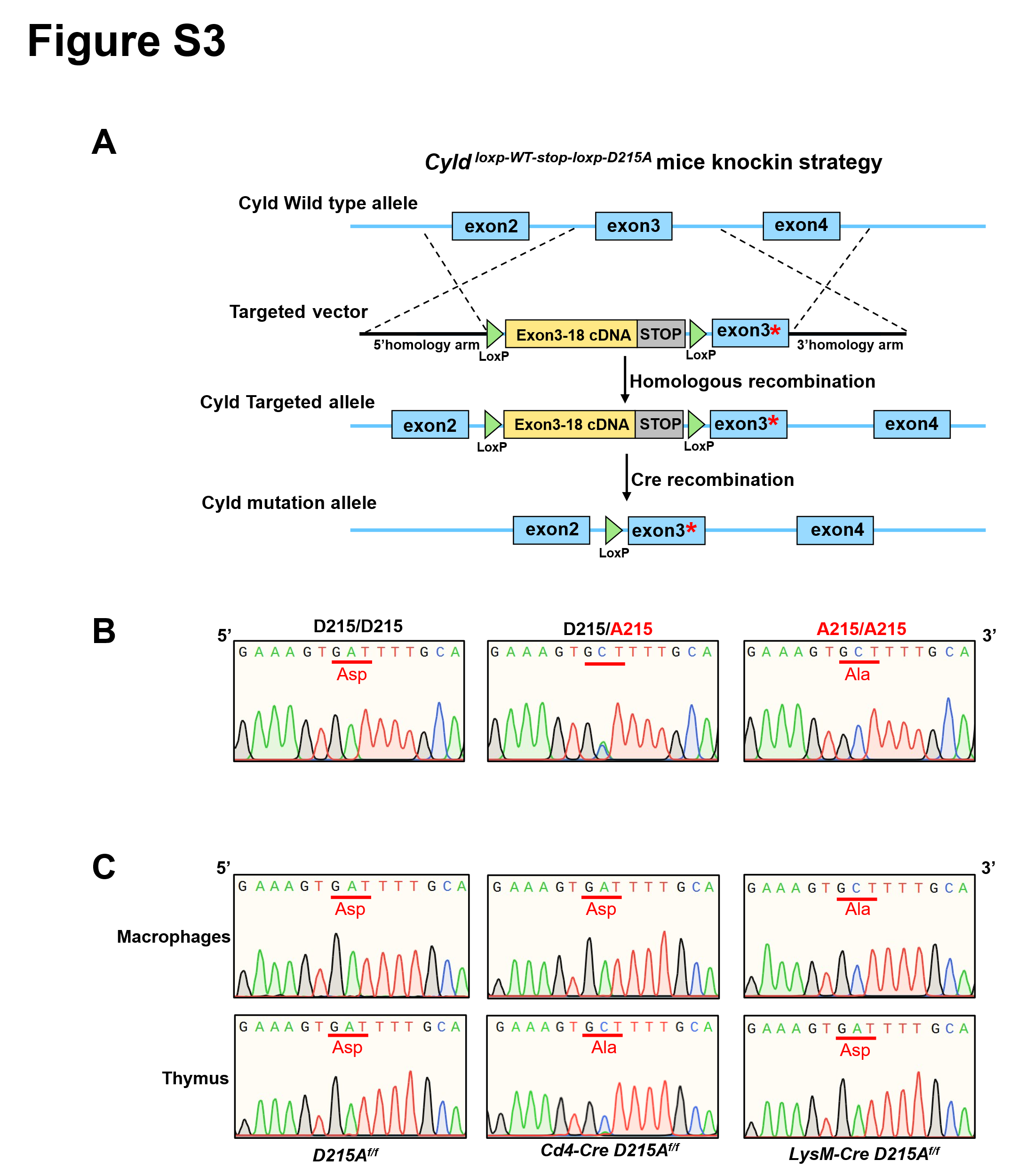
****Figure S3.** Generation and validation of conditional *Cyld^D215A^* knock-in mice. A) Schematic overview of strategy to generate conditional *Cyld^D215A^* knock-in *Cyld ^loxp-WT-stop-loxp-D215A^* mice (*D215A^f/f^*). B) Genotyping validation showing mutation of aspartic acid to alanine at position 215 (D215A) in CYLD, with PCR analysis of toe DNA using allele-specific primers and Sanger sequencing confirmation. C) Peritoneal macrophages and thymic tissue (T cell–enriched) were isolated from indicated genotypes, the Cyld region spanning codon 215 was amplified from cell-type–specific cDNA and analyzed by sequencing.

**
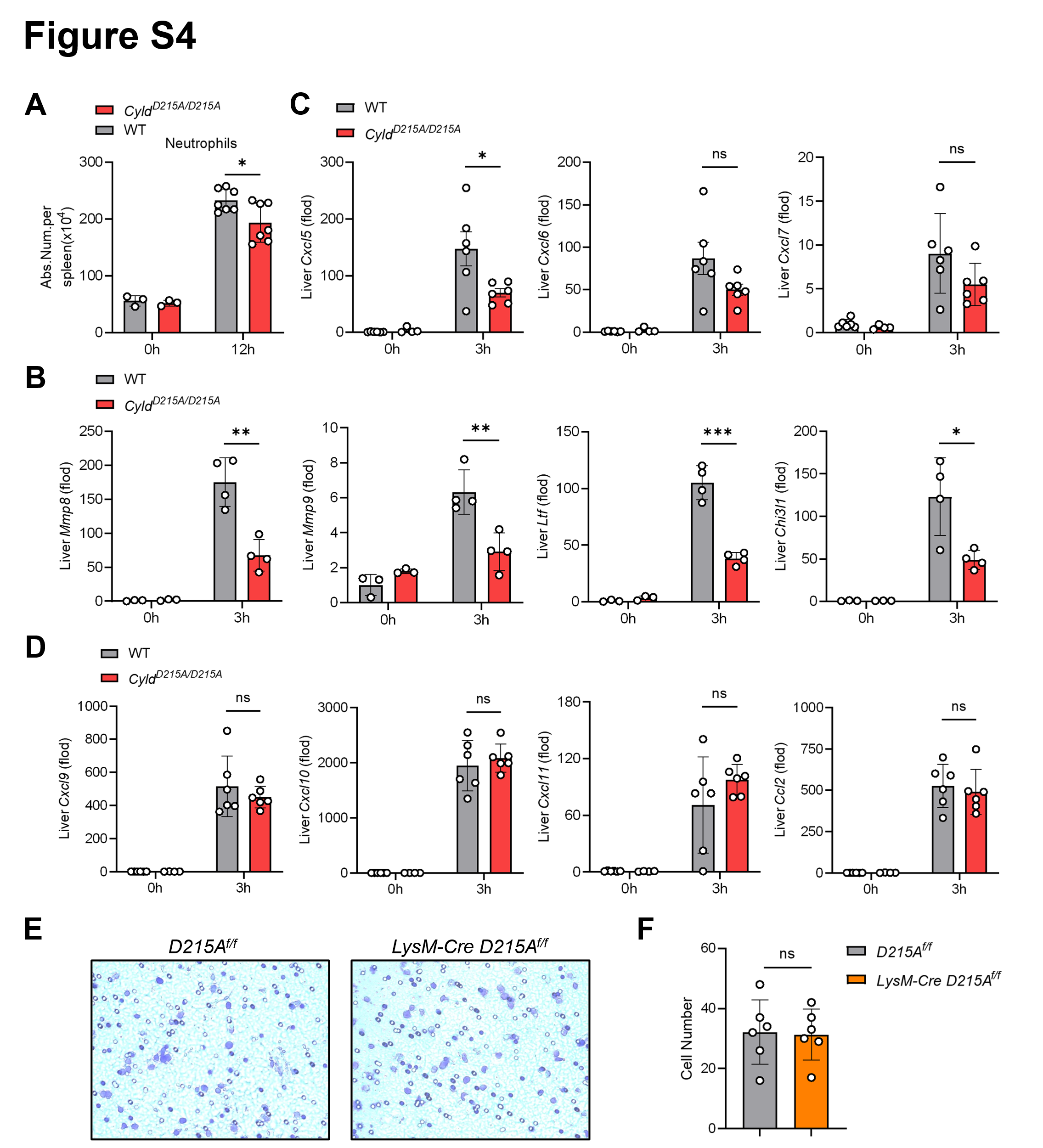
Figure S4.** Analysis of neutrophil accumulation, chemokine expression, and chemotactic responsiveness. A) Flow cytometry analysis of splenocytes from indicated genotypes after ConA treatment (n = 3 mice per group for untreated controls and n =7 mice per group for ConA-treated groups). B–D) RT-qPCR analysis of neutrophils marker genes (*Mmp8*, *Mmp9*, *Ltf*, *Chi3li*), neutrophil-recruiting chemokines (*Cxcl5*, *Cxcl6*, *Cxcl7*), lymphocyte-recruiting chemokines (*Cxcl9*, *Cxcl10*) and monocyte-recruiting chemokine (*Ccl2*) expression in liver tissues from mice of indicated genotypes 3 h after ConA treatment (12 mg/kg, i.v.) (n ≥ 3 mice per group for untreated controls and n ≥ 4 mice per group for ConA-treated groups).. E, F) Bone marrow–derived neutrophils from the indicated genotypes were analyzed using Transwell assays. Neutrophils were placed in the upper chamber containing serum-free RPMI 1640, and mouse CXCL1 (100 ng/mL) in serum-free RPMI 1640 was added to the lower chamber. After 6 h, neutrophils that traversed the membrane and were present on the lower surface of the insert were fixed and stained with crystal violet for analysis. (E) Representative images of the lower membrane surface are shown (40×). (F) Quantification of transmigrated neutrophils. Statistical analysis was performed using a two-tailed unpaired Student's *t*-test. Data are presented as mean ± SD, **P* < 0.05, ***P* < 0.01, ****P* < 0.001, *****P* < 0.0001; ns, not significant.

**
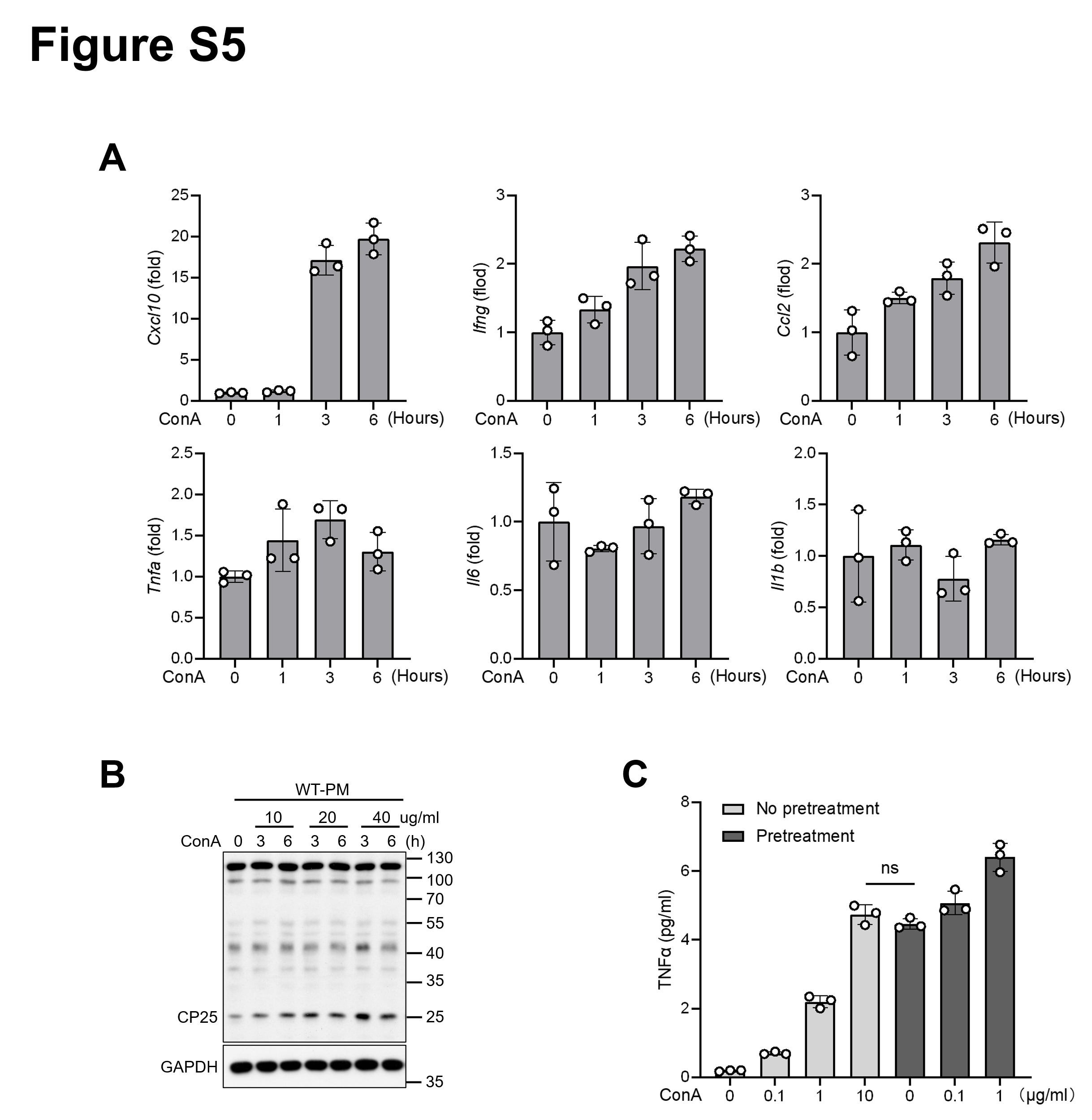
Figure S5.** Effects of ConA on TNFα production. A) RT-qPCR analysis of WT PMs stimulated with ConA (10 μg/mL) for indicated times (n = 3 biological replicates). B) WT PMs were treated with ConA at the indicated concentrations for the indicated durations, and cell lysates were analyzed by immunoblotting. C) T cells and macrophages were directly co-cultured in the presence of increasing concentrations of ConA (0.1, 1, or 10 μg/mL) plus Polymyxin B (25 μg/mL) for 6h, and TNFα levels in the supernatants were measured as a functional readout (no pretreatment). In parallel, macrophages were pretreated with ConA (10 μg/mL) for 3 h, washed, and subsequently co-cultured with T cells in the presence of the indicated ConA concentrations for 6h, followed by TNFα quantification (pretreatment) (n = 3 biological replicates). Statistical analysis for panels C was performed using a two-tailed unpaired Student's *t*-test. Data are presented as mean ± SD, **P* < 0.05; ns, not significant.

**
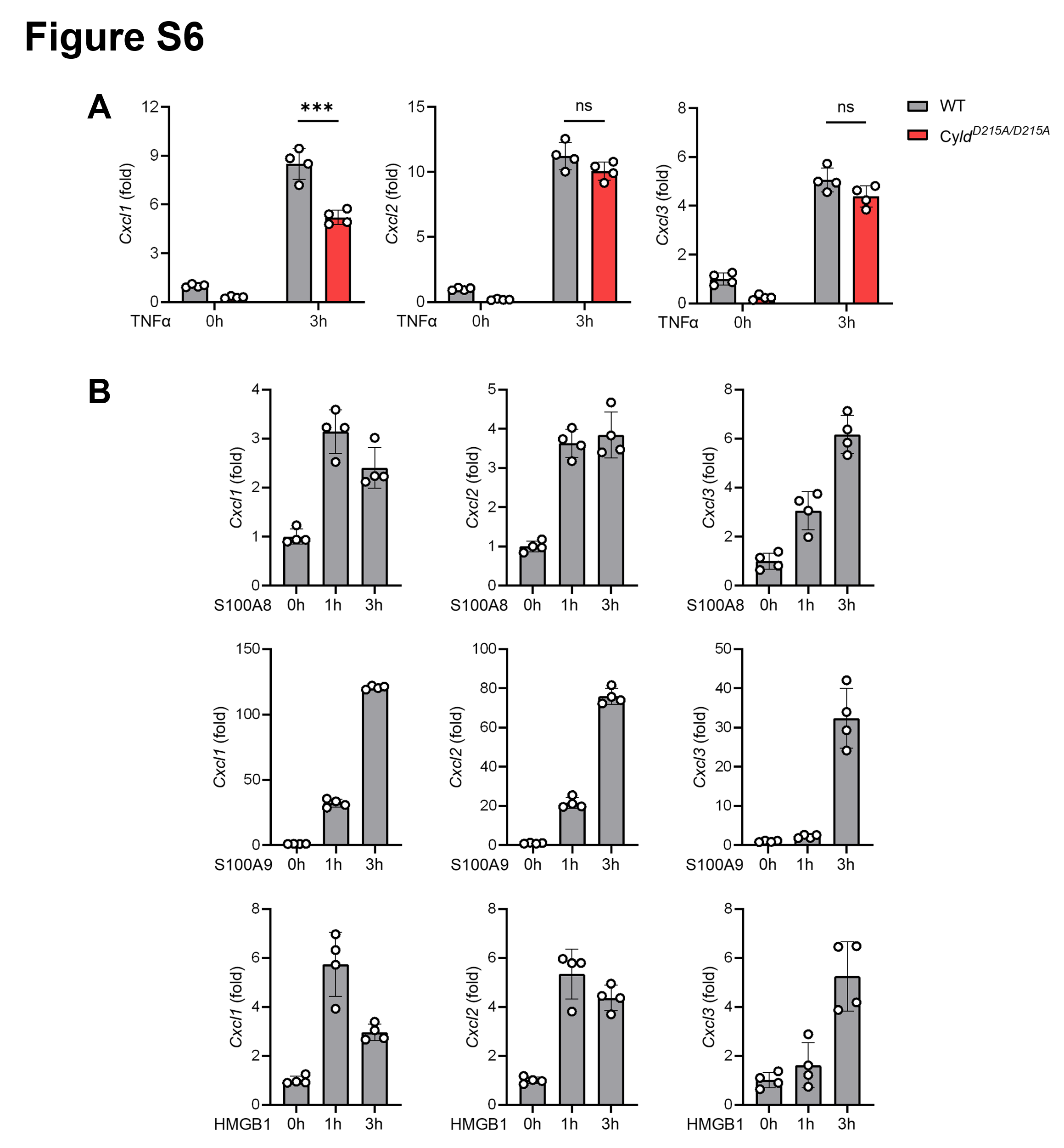
Figure S6.** S100A9 demonstrates potent chemokine-inducing activity. A) RT-qPCR analysis of PMs from the indicated mice stimulated with mouse TNFα (20 ng/mL) for indicated times (n = 4 biological replicates). B) RT-qPCR analysis of WT PMs stimulated with S100A8 (400 ng/mL), S100A9 (400 ng/mL), or HMGB1 (400 ng/mL) for the indicated times (n = 4 biological replicates). Statistical analysis for panels A was performed using a two-tailed unpaired Student's *t*-test. Data are presented as mean ± SD, **P* < 0.05, ***P* < 0.01, ****P* < 0.001, *****P* < 0.0001; ns, not significant.

**
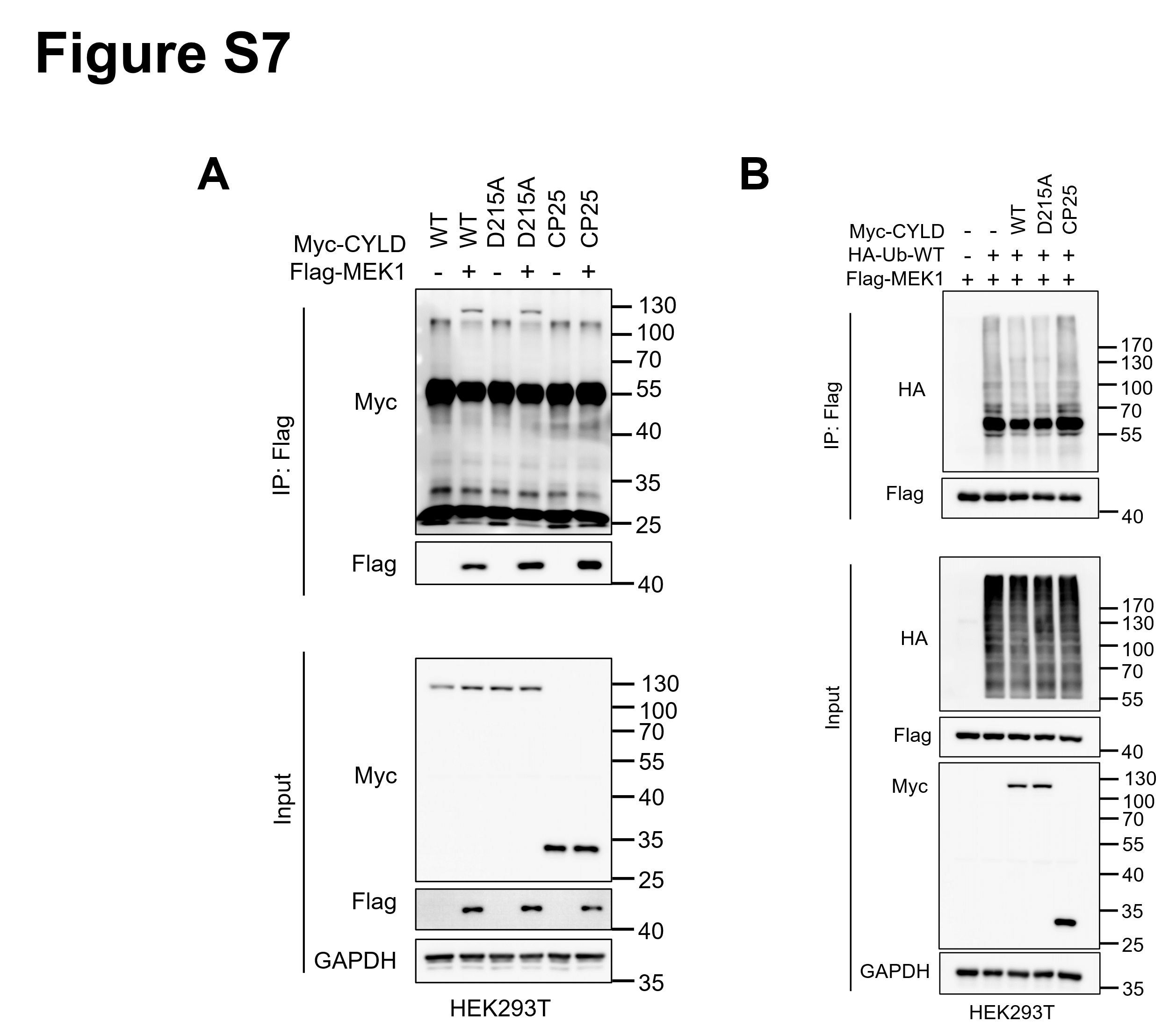
****Figure S7.** The CP25 fragment dose not regulate MEK1. A, B) HEK293T cells were transfected with the indicated plasmids or corresponding empty vectors for 24 h. Cell lysates were subjected to immunoprecipitation with anti-Flag beads and immunoblot analysis.

**
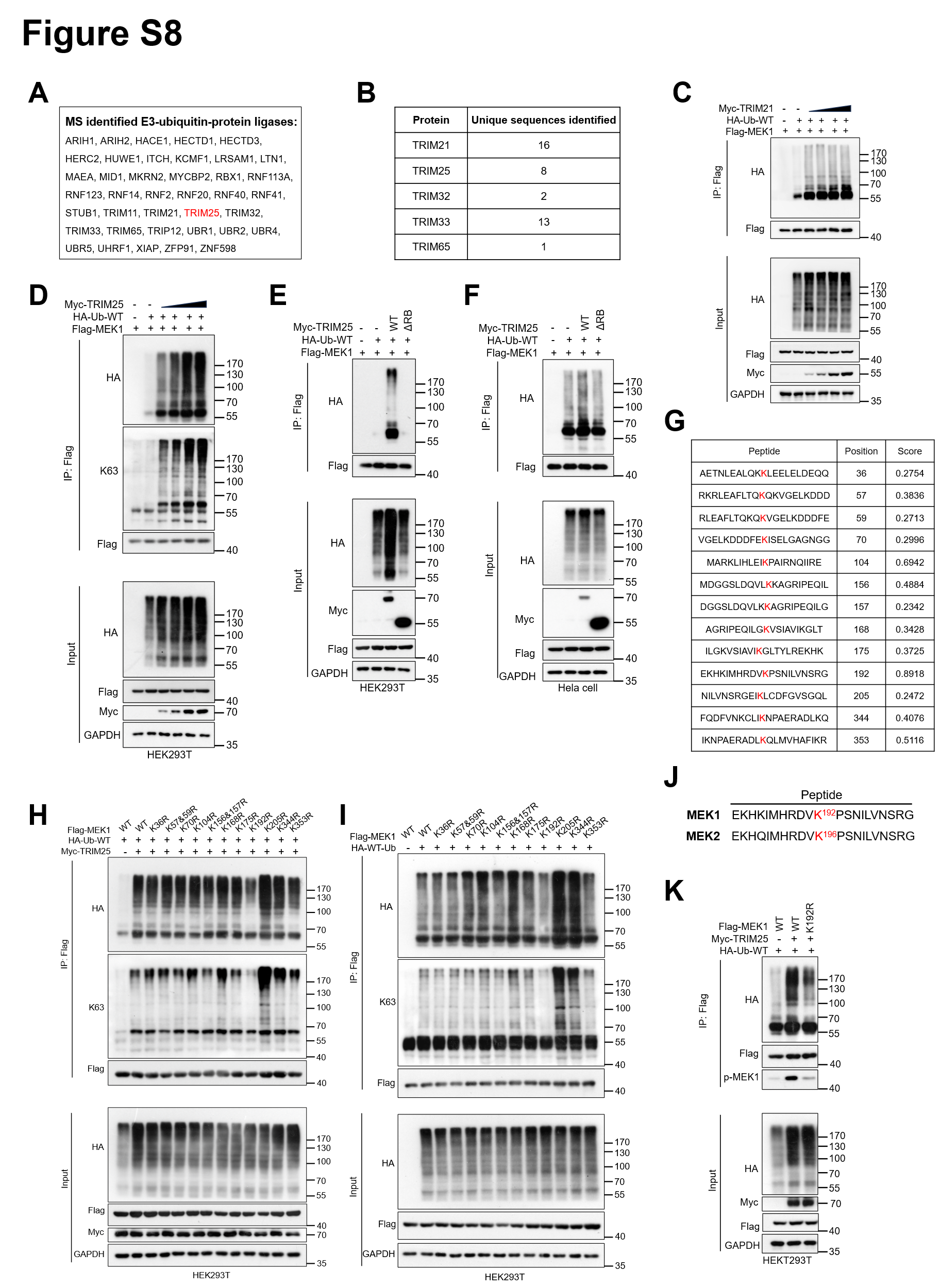
****Figure S8.** CYLD coordinating with TRIM25 regulates K63-linked ubiquitination of MEK1/2 on K192/K196 residues. A) Mass spectrometry analysis of HEK293T cells transfected with Flag-MEK1. Flag-MEK1 complexes were immunoprecipitated with anti-Flag beads, and potential MEK1-interacting E3 ubiquitin ligases were identified. B) Numbers of ubiquitin-modified peptide sequences identified per protein by mass spectrometry. C) HEK293T cells were transfected with Flag-MEK1, HA-Ub-WT, and Myc-TRIM21 (increasing doses) with the indicated combinations for 24 h. Cell lysates were subjected to immunoprecipitation with anti-Flag beads and immunoblotted with the indicated antibodies. D) HEK293T cells were transfected with Flag-MEK1, HA-Ub-WT, and Myc-TRIM25 (increasing doses) with the indicated combinations for 24 h. Cell lysates were subjected to immunoprecipitation with anti-Flag beads and immunoblotted with the indicated antibodies. E, F) HEK293T cells (E) and HeLa cells (F) were transfected with Flag-MEK1, HA-Ub-WT, and Myc-TRIM25 (or mutants) with the indicated combinations for 24 h. Cell lysates were subjected to immunoprecipitation with anti-Flag beads and immunoblotted with the indicated antibodies. G) Predicted ubiquitination sites on murine MEK1/2 (shown in red) using the GPS-Uber prediction tool ([GPS-Uber - Ubiquitin-protein ligase enzymes-substrate relationship prediction](https://gpsuber.biocuckoo.cn/)). (H) HEK293T cells were transfected with Flag-MEK1 (or mutants), HA-Ub-WT, and Myc-TRIM25 with the indicated combinations for 24 h. Cell lysates were subjected to immunoprecipitation with anti-Flag beads and immunoblotted with the indicated antibodies. I) HEK293T cells were transfected with Flag-MEK1 (or mutants) and HA-Ub-WT with the indicated combinations for 24 h. Cell lysates were subjected to immunoprecipitation with anti-Flag beads and immunoblotted with the indicated antibodies. J) Sequence alignment of mouse MEK1 and MEK2 showing conserved ubiquitination sites (red). K) HEK293T cells were transfected with Flag-MEK1 (or mutants), HA-Ub-WT, and Myc-TRIM25 with the indicated combinations for 24 h. Cell lysates were subjected to immunoprecipitation with anti-Flag beads and immunoblotted with the indicated antibodies.

**
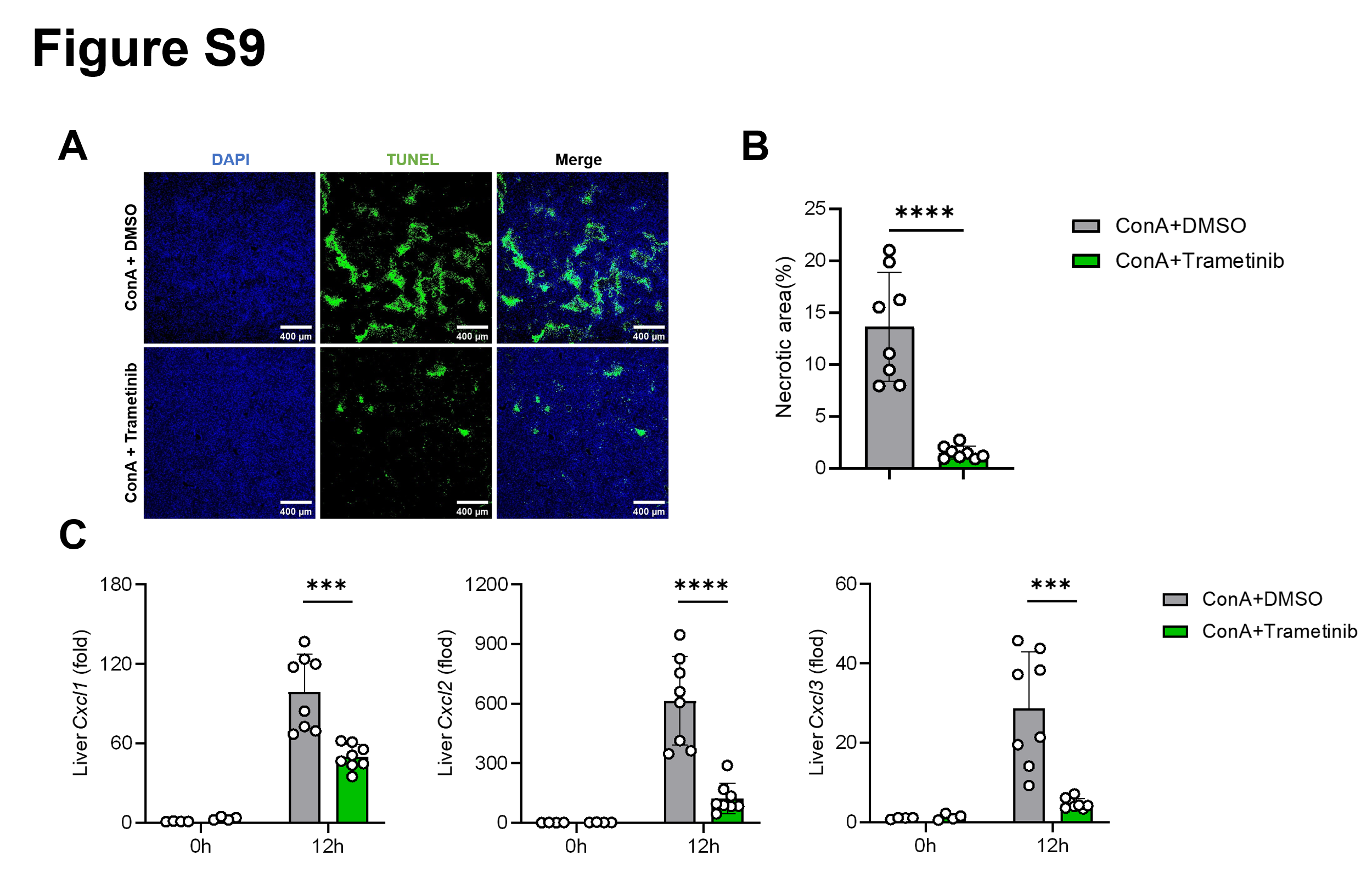
Figure S9.** pharmacological inhibition of MEK1/2 attenuates EAH severity. A–C) WT mice were pretreated with trametinib (5 mg/kg, i.p.) or vehicle 2 h before ConA challenge (12 mg/kg, i.v.) (n = 8 mice per group). Liver tissues collected at 12 h were analyzed for: (A) TUNEL staining of liver sections (scale bar: 400 μm), (B) Quantification of TUNEL-positive areas, and (C) RT-qPCR analysis of neutrophil-recruiting chemokine genes (*Cxcl1*, *Cxcl2* and *Cxcl3*.). Statistical analysis for panels B and C was performed using a two-tailed unpaired Student's *t*-test. Data are presented as mean ± SD, **P* < 0.05, ***P* < 0.01, ****P* < 0.001, *****P* < 0.0001.

**Table S1**

| Gene | Forward primer (5'-3') | Reverse primer (5'-3') |
| --- | --- | --- |
| *Cyld* | ACCCTACTGGGAAGAACGGAT | CGGTCTTGGATGTACTGTCCTAT |
| *Cxcl1* | CTGGGATTCACCTCAAGAACATC | CAGGGTCAAGGCAAGCCTC |
| *Cxcl2* | CCAACCACCAGGCTACAGG | GCGTCACACTCAAGCTCTG |
| *Cxcl3* | TGAGACCATCCAGAGCTTGACG | CCTTGGGGGTTGAGGCAAACTT |
| *Cxcl5* | GGTCCACAGTGCCCTACG | GCGAGTGCATTCCGCTTA |
| *Cxcl6* | TGGATCCAGAAGCTCCTGTGA | TGCATTCCGCTTAGCTTTCTTT |
| *Cxcl7* | TGGGCCTGATCCTTGTTGCGC | GCACCGTTTTTTGTCCATTCTTCAG |
| *Cxcl9* | GGAGTTCGAGGAACCCTAGTG | GGGATTTGTAGTGGATCGTGC |
| *Cxcl10* | CCCACGTGTTGAGATCATTG | CACTGGGTAAAGGGGAGTGA |
| *Cxcl11* | CCGAGTAACGGCTGCGACAAAG | CCTGCATTATGAGGCGAGCTTG |
| *Ly6g* | GACTTCCTGCAACACAACTACC | ACAGCATTACCAGTGATCTCAGT |
| *Mmp8* | TCTTCCTCCACACACAGCTTG | CTGCAACCATCGTGGCATTC |
| *Mmp9* | CTGGACAGCCAGACACTAAAG | CTCGCGGCAAGTCTTCAGAG |
| *Ltf* | TGAGGCCCTTGGACTCTGT | ACCCACTTTTCTCATCTCGTTC |
| *Lcn2* | TGGCCCTGAGTGTCATGTG | CTCTTGTAGCTCATAGATGGTGC |
| *Chi3l1* | GTACAAGCTGGTCTGCTACTTC | ATGTGCTAAGCATGTTGTCGC |
| *Ccl2* | CCAGCCTACTCATTGGGAT | GGGCCTGCTGTTCACAGTT |
| *Actb* | GTGGGGCGCCCCAGGCACCA | CTCCTTAATGTCACGCACGATTTC |
| *Rn18s* | AACTTTCGATGGTAGTCGCCGT | TCCTTGGATGTGGTAGCCGTTT |

**Table S1.** qPCR primer sequences for *Mus musculus* genes.
